# Development and validation of a prognostic model for early triage of patients diagnosed with COVID-19

**DOI:** 10.1038/s41598-021-01452-7

**Published:** 2021-11-09

**Authors:** Chansik An, Hyun Cheol Oh, Jung Hyun Chang, Seung-Jin Oh, Jung Mo Lee, Chang Hoon Han, Seong Woo Kim

**Affiliations:** 1 Research Institute, National Health Insurance Service Ilsan Hospital, Goyang, Korea; 2 Department of Radiology, National Health Insurance Service Ilsan Hospital, Goyang, Korea; 3 Department of Orthopedic Surgery, National Health Insurance Service Ilsan Hospital, Goyang, Korea; 4 Department of Otolaryngology-Head and Neck Surgery, National Health Insurance Service Ilsan Hospital, Goyang, Korea; 5 Division of Cardiology, Department of Internal Medicine, National Health Insurance Service Ilsan Hospital, Goyang, Korea; 6 Division of Pulmonology, Department of Internal Medicine, National Health Insurance Service Ilsan Hospital, Goyang, Korea; 7 Department of Physical Medicine and Rehabilitation, National Health Insurance Service Ilsan Hospital, Goyang, Korea

**Keywords:** Viral infection, Nomograms

## Abstract

We developed a tool to guide decision-making for early triage of COVID-19 patients based on a predicted prognosis, using a Korean national cohort of 5,596 patients, and validated the developed tool with an external cohort of 445 patients treated in a single institution. Predictors chosen for our model were older age, male sex, subjective fever, dyspnea, altered consciousness, temperature ≥ 37.5 °C, heart rate ≥ 100 bpm, systolic blood pressure ≥ 160 mmHg, diabetes mellitus, heart disease, chronic kidney disease, cancer, dementia, anemia, leukocytosis, lymphocytopenia, and thrombocytopenia. In the external validation, when age, sex, symptoms, and underlying disease were used as predictors, the AUC used as an evaluation metric for our model’s performance was 0.850 in predicting whether a patient will require at least oxygen therapy and 0.833 in predicting whether a patient will need critical care or die from COVID-19. The AUCs improved to 0.871 and 0.864, respectively, when additional information on vital signs and blood test results were also used. In contrast, the protocols currently recommended in Korea showed AUCs less than 0.75. An application for calculating the prognostic score in COVID-19 patients based on the results of this study is presented on our website (https://nhimc.shinyapps.io/ih-psc/), where the results of the validation ongoing in our institution are periodically updated.

## Introduction

Since the World Health Organization (WHO) declared the coronavirus disease 2019 (COVID-19) a pandemic in March 2020, it has been raging on, taking the lives of many people (over 4.4 million as of Aug 24, 2021)^1^. Since effective vaccines were recently developed, more than 5 billion doses have been administered worldwide. Still, however, more than 5 million people are being diagnosed with COVID-19 every week^1^. The treatment of COVID-19 mainly relies on symptomatic relief and supportive care, oxygen therapy, and critical care, depending on the disease severity. Thus, it is crucial to triage COVID-19 patients rapidly and efficiently so that limited medical resources, including quarantine facilities, hospital beds, and critical care equipment, can be allocated appropriately.

The current protocols recommended for triage and referral of COVID-19 patients in many countries or by WHO are based on known risk factors and expert opinion but have not been validated on the actual patient data^2–5^. Furthermore, since sudden disease progression in initially mild or asymptomatic COVID-19 patients is not rare with reported incidences of 6–12%^6–9^, we should base the triage and referral of COVID-19 patients on the worst severity expected during the disease course, rather than the severity at the time of diagnosis.

Data about the pandemic has now accumulated sufficiently to enable development of a data-driven prediction model for patient triage decision-making. Several prediction models for disease severity in COVID-19 patients have been proposed^10–20^. There may be limitations, however, to applying these models for COVID-19 patient triage under some real-world circumstances. Most of these models require patients’ information obtained from a blood test or imaging study. However, we often need to triage and refer COVID-19 patients immediately after the diagnosis with limited information depending on the situation.

Therefore, we aimed to develop and validate an easy-to-use tool for COVID-19 patient triage based on a predicted prognosis, with the flexibility to adapt to variable availability. We categorized variables into four groups—demographics and symptoms, underlying diseases, vital signs, and laboratory findings—and develop separate algorithms for different combinations of the variable groups. We compared the performance of our models with the currently used triage protocols. Lastly, we validated the final model in an independent external cohort.

## Results

### Patients

Of the total 5,596 COVID-19 patients in the model development cohort, approximately half of the patients (52.1%) were 50 years or older, while people aged younger than 20 years accounted for only 4.9% (Table 1). The two most common age groups were 20–29 years (19.8%) and 50–59 years (20.4%). The ratio of males to females was 5.9:4.1. Most (85.4%) recovered without particular therapy, 9.1% of the patients required oxygen therapy, and the remaining 5.4% fell into severe conditions such as respiratory or multi-organ failure and required critical care such as mechanical ventilation or extracorporeal membrane oxygenation (ECMO). The overall mortality from COVID-19 infection was 1.1% (63/5,596). The mean time between diagnosis and recovery or death was 25.6 days, with a standard deviation (SD) of 11.0 days. Patients who were older, male, under-weight or obese, or with symptoms (except for diarrhea), underlying diseases (except for autoimmune disease), abnormal vital signs (except for diastolic blood pressure), or abnormal blood test results tended to fall into more severe conditions (Table 1). The training and internal validation subcohorts comprised 3940 and 1656 patients, respectively. There was no significant difference in variables between the two subcohorts (Supplementary Table S1).

**Table 1:** Patient characteristics in the model development cohort by the worst severity during the disease course.

Variable	Supportive care	O_2_ therapy	Critical care	Mortality	*p* value	Total
**Number of patients**	4780 (85.4%)	512 (9.1%)	241 (4.3%)	63 (1.1%)	< 0.001	5596 (100%)
**Days to recovery or death, mean (SD)**	25.5 (10.3)	30.0 (12.1)	36.3 (16.1)	15.2 (13.4)	< 0.001	25.6 (11.0)
**Age**						
0–9 years	66 (100%)	0 (0%)	0 (0%)	0 (0%)	< 0.001	66 (100%)
10–19 years	203 (99%)	1 (0.5%)	0 (0%)	1 (0.5%)		205 (100%)
20–29 years	1087 (98%)	20 (1.8%)	0 (0%)	2 (0.2%)		1109 (100%)
30–39 years	546 (97.2%)	11 (2%)	2 (0.4%)	3 (0.5%)		562 (100%)
40–49 years	703 (95.1%)	34 (4.6%)	2 (0.3%)	0 (0%)		739 (100%)
50–59 years	999 (87.6%)	114 (10%)	15 (1.3%)	12 (1.1%)		1140 (100%)
60–69 years	713 (78.7%)	135 (14.9%)	34 (3.8%)	24 (2.6%)		906 (100%)
70–79 years	331 (60.7%)	125 (22.9%)	73 (13.4%)	16 (2.9%)		545 (100%)
≥ 80 years	132 (40.7%)	72 (22.2%)	115 (35.5%)	5 (1.5%)		324 (100%)
**Sex**						
Female	2858 (86.9%)	288 (8.8%)	114 (3.5%)	29 (0.9%)	< 0.001	3289 (100%)
Male	1922 (83.3%)	224 (9.7%)	127 (5.5%)	34 (1.5%)		2307 (100%)
**Pregnancy**						
No	4752 (85.3%)	512 (9.2%)	241 (4.3%)	63 (1.1%)	0.353	5568 (100%)
Yes	19 (100%)	0 (0%)	0 (0%)	0 (0%)		19 (100%)
Missing	9 (100%)	0 (0%)	0 (0%)	0 (0%)		9 (100%)
**Body mass index (kg/cm**^**2**^**)**						
< 18.5	225 (86.9%)	16 (6.2%)	16 (6.2%)	2 (0.8%)	< 0.001	259 (100%)
18.5–23	1660 (89.5%)	127 (6.9%)	46 (2.5%)	21 (1.1%)		1854 (100%)
23–25	893 (86.4%)	108 (10.5%)	20 (1.9%)	12 (1.2%)		1033 (100%)
25–30	865 (82.8%)	125 (12%)	39 (3.7%)	16 (1.5%)		1045 (100%)
> 30	178 (86%)	20 (9.7%)	5 (2.4%)	4 (1.9%)		207 (100%)
Missing	959 (80.1%)	116 (9.7%)	115 (9.6%)	8 (0.7%)		1198 (100%)
**Subjective fever**						
Absent	3818 (88.9%)	296 (6.9%)	148 (3.4%)	32 (0.7%)	< 0.001	4294 (100%)
Present	962 (73.9%)	216 (16.6%)	93 (7.1%)	31 (2.4%)		1302 (100%)
**Cough**						
Absent	2836 (86.9%)	239 (7.3%)	160 (4.9%)	30 (0.9%)	< 0.001	3265 (100%)
Present	1944 (83.4%)	273 (11.7%)	81 (3.5%)	33 (1.4%)		2331 (100%)
**Sputum**						
Absent	3456 (86.7%)	319 (8%)	169 (4.2%)	41 (1%)	< 0.001	3985 (100%)
Present	1324 (82.2%)	193 (12%)	72 (4.5%)	22 (1.4%)		1611 (100%)
**Dyspnea**						
Absent	4445 (90.1%)	332 (6.7%)	128 (2.6%)	26 (0.5%)	< 0.001	4931 (100%)
Present	335 (50.4%)	180 (27.1%)	113 (17%)	37 (5.6%)		665 (100%)
**Sore throat**						
Absent	3989 (84.4%)	446 (9.4%)	228 (4.8%)	61 (1.3%)	< 0.001	4724 (100%)
Present	791 (90.7%)	66 (7.6%)	13 (1.5%)	2 (0.2%)		872 (100%)
**Rhinorrhea**						
Absent	4216 (84.7%)	468 (9.4%)	235 (4.7%)	60 (1.2%)	< 0.001	4979 (100%)
Present	564 (91.4%)	44 (7.1%)	6 (1%)	3 (0.5%)		617 (100%)
**Myalgia**						
Absent	4005 (85.6%)	400 (8.6%)	220 (4.7%)	52 (1.1%)	< 0.001	4677 (100%)
Present	775 (84.3%)	112 (12.2%)	21 (2.3%)	11 (1.2%)		919 (100%)
**Fatigue**						
Absent	4606 (85.9%)	475 (8.9%)	224 (4.2%)	58 (1.1%)	< 0.001	5363 (100%)
Present	174 (74.7%)	37 (15.9%)	17 (7.3%)	5 (2.1%)		233 (100%)
**Headache**						
Absent	3931 (84.8%)	421 (9.1%)	228 (4.9%)	53 (1.1%)	< 0.001	4633 (100%)
Present	849 (88.2%)	91 (9.4%)	13 (1.3%)	10 (1%)		963 (100%)
**Nausea or vomiting**						
Absent	4598 (85.9%)	470 (8.8%)	225 (4.2%)	59 (1.1%)	< 0.001	5352 (100%)
Present	182 (74.6%)	42 (17.2%)	16 (6.6%)	4 (1.6%)		244 (100%)
**Diarrhea**						
Absent	4354 (85.7%)	446 (8.8%)	223 (4.4%)	57 (1.1%)	0.022	5080 (100%)
Present	426 (82.6%)	66 (12.8%)	18 (3.5%)	6 (1.2%)		516 (100%)
**Altered consciousness**						
Absent	4772 (85.8%)	512 (9.2%)	218 (3.9%)	59 (1.1%)	< 0.001	5561 (100%)
Present	8 (22.9%)	0 (0%)	23 (65.7%)	4 (11.4%)		35 (100%)
**Diabetes mellitus**						
Absent	4322 (88%)	397 (8.1%)	143 (2.9%)	47 (1%)	< 0.001	4909 (100%)
Present	458 (66.7%)	115 (16.7%)	98 (14.3%)	16 (2.3%)		687 (100%)
**Hypertension**						
Absent	3966 (90.2%)	304 (6.9%)	97 (2.2%)	31 (0.7%)	< 0.001	4398 (100%)
Present	814 (67.9%)	208 (17.4%)	144 (12%)	32 (2.7%)		1198 (100%)
**Heart disease**						
Absent	4756 (85.9%)	497 (9%)	223 (4%)	61 (1.1%)	< 0.001	5537 (100%)
Present	24 (40.7%)	15 (25.4%)	18 (30.5%)	2 (3.4%)		59 (100%)
**Asthma**						
Absent	4682 (85.6%)	495 (9.1%)	228 (4.2%)	63 (1.2%)	0.001	5468 (100%)
Present	98 (76.6%)	17 (13.3%)	13 (10.2%)	0 (0%)		128 (100%)
**Chronic obstructive pulmonary disease**						
Absent	4760 (85.7%)	502 (9%)	233 (4.2%)	61 (1.1%)	< 0.001	5556 (100%)
Present	20 (50%)	10 (25%)	8 (20%)	2 (5%)		40 (100%)
**Chronic kidney disease**						
Absent	4757 (85.9%)	498 (9%)	225 (4.1%)	61 (1.1%)	< 0.001	5541 (100%)
Present	23 (41.8%)	14 (25.5%)	16 (29.1%)	2 (3.6%)		55 (100%)
**Cancer**						
Absent	4679 (85.8%)	490 (9%)	219 (4%)	63 (1.2%)	< 0.001	5451 (100%)
Present	101 (69.7%)	22 (15.2%)	22 (15.2%)	0 (0%)		145 (100%)
**Chronic liver disease**						
Absent	4404 (84.9%)	490 (9.4%)	234 (4.5%)	62 (1.2%)	0.033	5190 (100%)
Present	61 (73.5%)	14 (16.9%)	7 (8.4%)	1 (1.2%)		83 (100%)
missing	315 (97.5%)	8 (2.5%)	0 (0%)	0 (0%)		323 (100%)
**Autoimmune disease**						
Absent	4430 (84.7%)	498 (9.5%)	238 (4.6%)	63 (1.2%)	0.356	5229 (100%)
Present	29 (76.3%)	6 (15.8%)	3 (7.9%)	0 (0%)		38 (100%)
missing	321 (97.6%)	8 (2.4%)	0 (0%)	0 (0%)		329 (100%)
**Dementia**						
Absent	4355 (86.3%)	463 (9.2%)	166 (3.3%)	62 (1.2%)	< 0.001	5046 (100%)
Present	107 (47.8%)	41 (18.3%)	75 (33.5%)	1 (0.4%)		224 (100%)
missing	318 (97.5%)	8 (2.5%)	0 (0%)	0 (0%)		326 (100%)
**Heart rate (beat/min)**						
Bradycardia (< 60)	87 (80.6%)	15 (13.9%)	6 (5.6%)	0 (0%)	0.001	108 (100%)
Normal (60–100)	3799 (86.3%)	394 (8.9%)	160 (3.6%)	50 (1.1%)		4403 (100%)
Tachycardia (> 100)	784 (81.8%)	102 (10.6%)	61 (6.4%)	12 (1.3%)		959 (100%)
Missing	110 (87.3%)	1 (0.8%)	14 (11.1%)	1 (0.8%)		126 (100%)
**Body temperature (°C)**						
< 37.5	4300 (88.0%)	380 (7.8%)	166 (3.4%)	39 (0.8%)	< 0.001	4885 (100%)
37.5–38	349 (75.4%)	70 (15.1%)	36 (7.8%)	8 (1.7%)		463 (100%)
38–38.5	74 (54.4%)	31 (22.8%)	21 (15.4%)	10 (7.4%)		136 (100%)
38.5 ≥ 38.5	32 (43.8%)	29 (39.7%)	6 (8.2%)	6 (8.2%)		73 (100%)
Missing	25 (64.1%)	2 (5.1%)	12 (30.8%)	0 (0%)		39 (100%)
**Systolic blood pressure (mmHg)**						
< 120	1140 (87.3%)	95 (7.3%)	58 (4.4%)	13 (1%)	< 0.001	1306 (100%)
120–129	988 (86.8%)	110 (9.7%)	28 (2.5%)	12 (1.1%)		1138 (100%)
130–139	939 (86.7%)	101 (9.3%)	32 (3%)	11 (1%)		1083 (100%)
140–159	1190 (84%)	141 (10%)	68 (4.8%)	18 (1.3%)		1417 (100%)
≥ 160	402 (78.4%)	65 (12.7%)	37 (7.2%)	9 (1.8%)		513 (100%)
Missing	121 (87.1%)	0 (0%)	18 (12.9%)	0 (0%)		139 (100%)
**Diastolic blood pressure (mmHg)**						
< 80	1763 (83.9%)	208 (9.9%)	104 (4.9%)	27 (1.3%)	0.266	2102 (100%)
80–89	1557 (86.7%)	156 (8.7%)	61 (3.4%)	22 (1.2%)		1796 (100%)
90–99	907 (86%)	102 (9.7%)	36 (3.4%)	10 (0.9%)		1055 (100%)
≥ 100	432 (85.7%)	46 (9.1%)	22 (4.4%)	4 (0.8%)		504 (100%)
Missing	121 (87.1%)	0 (0%)	18 (12.9%)	0 (0%)		139 (100%)
**Hemoglobin (g/dL)**						
Anemia	715 (69.8%)	162 (15.8%)	128 (12.5%)	20 (2%)	< 0.001	1025 (100%)
Normal*	2137 (84.7%)	267 (10.6%)	87 (3.4%)	32 (1.3%)		2523 (100%)
Elevated	471 (88.5%)	40 (7.5%)	14 (2.6%)	7 (1.3%)		532 (100%)
Missing	1457 (96.1%)	43 (2.8%)	12 (0.8%)	4 (0.3%)		1516 (100%)
**Hematocrit (%)**						
Anemia	576 (66.1%)	151 (17.3%)	124 (14.2%)	21 (2.4%)	< 0.001	872 (100%)
Normal**	2235 (85%)	274 (10.4%)	90 (3.4%)	31 (1.2%)		2630 (100%)
Elevated	505 (88.1%)	45 (7.9%)	16 (2.8%)	7 (1.2%)		573 (100%)
Missing	1464 (96.3%)	42 (2.8%)	11 (0.7%)	4 (0.3%)		1521 (100%)
**White blood cell count (× 10**^**3**^**/µL)**						
Leukocytopenia (< 4)	555 (80.6%)	99 (14.4%)	27 (3.9%)	8 (1.2%)	< 0.001	689 (100%)
Normal (4–11)	2628 (83.3%)	336 (10.7%)	149 (4.7%)	41 (1.3%)		3154 (100%)
Leukocytosis (≥ 11)	141 (59.2%)	35 (14.7%)	53 (22.3%)	9 (3.8%)		238 (100%)
Missing	1456 (96.1%)	42 (2.8%)	12 (0.8%)	5 (0.3%)		1515 (100%)
**Lymphocyte count (× 10**^**3**^**/µL)**						
Lymphocytopenia (< 1)	407 (51.8%)	196 (25%)	147 (18.7%)	35 (4.5%)	< 0.001	785 (100%)
Normal (1–4.8)	2871 (88.7%)	267 (8.2%)	77 (2.4%)	23 (0.7%)		3238 (100%)
Lymphocytosis (> 4.8)	33 (100%)	0 (0%)	0 (0%)	0 (0%)		33 (100%)
Missing	1469 (95.4%)	49 (3.2%)	17 (1.1%)	5 (0.3%)		1540 (100%)
**Platelet count (× 10**^**3**^**/µL)**						
Thrombocytopenia (< 150)	294 (58.8%)	106 (21.2%)	85 (17%)	15 (3%)	< 0.001	500 (100%)
Normal (150–450)	2971 (84.7%)	352 (10%)	142 (4%)	44 (1.3%)		3509 (100%)
Thrombocytosis (> 450)	59 (81.9%)	11 (15.3%)	2 (2.8%)	0 (0%)		72 (100%)
Missing	1456 (96.1%)	43 (2.8%)	12 (0.8%)	4 (0.3%)		1515 (100%)

Values in cells and parentheses are the number and percentage of patients, respectively, except for the days to recovery or death. Patient characteristics in the external validation cohort is summarized in Supplementary Table S2.

*Male, 13.8–17.2 g/dL; Female, 12.1–15.1 g/dL.

**Male, 41–50%; Female, 36–48%.

In the external validation cohort of 445 patients, the mean age was 59 years (SD, 20 years) with the ratio of males to females of 4.7:5.3. Of these, 8.8% (39/445) required intensive treatment or died of COVID-19. The detailed characteristics are summarized in Supplementary Table S2.

### Selected predictors for each model

The full results of predictor selection are in Supplementary Table S3.

#### Model 1: from history taking

The predictors selected from Tier 1 variables for Model 1 were age, sex, and symptoms of subjective fever, rhinorrhea, dyspnea, and altered consciousness. As opposed to other selected predictors, rhinorrhea was associated with a better prognosis.

#### Model 2A: from history taking with known underlying disease status

The predictors chosen for Model2A were age, sex, subjective fever, dyspnea, and altered consciousness from Tier 1 (rhinorrhea excluded), and underlying diseases of hypertension, diabetes mellitus (DM), heart disease, chronic kidney disease (CKD), cancer, and dementia from Tier 2 variables.

#### Model 2B: from history taking and physical examination with uncertain underlying disease

The predictors were age, sex, subjective fever, rhinorrhea, dyspnea, and altered consciousness from Tier 1, and high body temperature and tachycardia from Tier 3 variables.

#### Model 3: from history taking and physical examination with known underlying disease status

The predictors were age, sex, subjective fever, dyspnea, and altered consciousness from Tier 1 (rhinorrhea not included), severe hypertension (systolic blood pressure ≥ 160 mmHg), DM, heart disease, CKD, cancer, and dementia from Tier 2, and high body temperature and tachycardia from Tier 3.

#### Model 4: on admission

The predictors were age, sex, subjective fever, dyspnea, and altered consciousness from Tier 1, severe hypertension, DM, heart disease, CKD, cancer, and dementia from Tier 2, and high body temperature from Tier 3 (tachycardia excluded), and anemia, leukocytosis, lymphocytopenia, and thrombocytopenia from Tier 4 variables.

### Variable effect size

**Figure 1:**
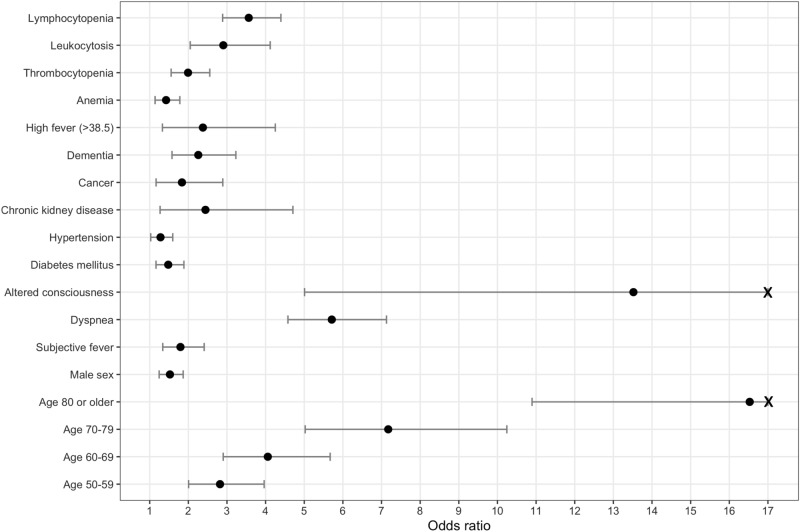
Forest plot showing the odds ratios of final predictors from multivariable ordinal logistic regression. The horizontal error bars indicated 95% confidence intervals. Note that the upper limit of 95% confidence interval is truncated for altered consciousness and age > 80. Detailed results are presented in Supplementary Table S4, including the odds ratios with confidence intervals from univariable and multivariable regression analyses.

Older age, altered consciousness, dyspnea, lymphocytopenia, leukocytosis, CKD, temperature of ≥ 38.5 °C, dementia, thrombocytopenia, cancer, subjective fever, male sex, anemia, DM were associated independently with prognosis, in decreasing order of odds ratio (OR) from the multivariable ordinal logistic regression (OLR) in the entire cohort (Fig. 1 and Supplementary Table S4).

### Model performance

#### Conventional protocols

In predicting whether a patient will require more than supportive care, the Korea Medical Association (KMA) model showed an area under the curve (AUC) of 0.723 (95% confidence interval [CI], 0.693–0.753) with a sensitivity of 54.9 (48.3–61.4)% and a specificity of 7.6 (6.3–9.1)%, and the AUC, sensitivity, and specificity of the Modified Early Warning Score (MEWS) were 0.598 (0.563–0.633), 56.8 (50.1–63.4)%, and 23.5 (21.2–25.9)%, respectively, in the internal validation cohort (Table 2).

**Table 2:** Model performance in early prediction of prognosis in COVID19 patients in the internal validation cohort.

Model	No significant treatment versus O_2_ therapy or more							No critical care required versus critical care* or death						
	AUC	TP/TN/FP/FN	Sensitivity	Specificity	Accuracy	Precision	NPV	AUC	TP/TN/FP/FN	Sensitivity	Specificity	Accuracy	Precision	NPV
**OLR**														
Model1	0.880 (0.855–0.904)	193/1199/236/48	80.1% (74.5–84.9)	83.6% (81.5–85.4)	83.1% (81.2–84.8)	45% (40.2–49.8)	96.2% (94.9–97.1)	0.903 (0.869–0.937)	75/1336/251/14	84.3% (75–91.1)	84.2% (82.3–85.9)	84.2% (82.4–85.9)	23% (18.5–28)	99% (98.3–99.4)
Model2A	0.889 (0.865–0.912)	195/1119/209/43	81.9% (76.4–86.6)	84.3% (82.2–86.2)	83.9% (82–85.7)	48.3% (43.3–53.3)	96.3% (95–97.3)	0.905 (0.869–0.940)	81/1164/313/8	91% (83.1–96)	78.8% (76.6–80.9)	79.5% (77.4–81.5)	20.6% (16.7–24.9)	99.3% (98.7–99.7)
Model2B	0.866 (0.841–0.892)	181/1147/261/53	77.4% (71.4–82.5)	81.5% (79.3–83.5)	80.9% (78.9–82.8)	41% (36.3–45.7)	95.6% (94.3–96.7)	0.914 (0.884–0.944)	72/1312/247/11	86.7% (77.5–93.2)	84.2% (82.2–85.9)	84.3% (82.4–86)	22.6% (18.1–27.6)	99.2% (98.5–99.6)
Model3	0.894 (0.871–0.917)	192/1082/210/40	82.8% (77.3–87.4)	83.7% (81.6–85.7)	83.6% (81.6–85.4)	47.8% (42.8–52.8)	96.4% (95.2–97.4)	0.922 (0.892–0.953)	76/1199/242/7	91.6% (83.4–96.5)	83.2% (81.2–85.1)	83.7% (81.7–85.5)	23.9% (19.3–29)	99.4% (98.8–99.8)
Model4	0.907 (0.884–0.929)	189/835/172/31	85.9% (80.6–90.2)	82.9% (80.5–85.2)	83.5% (81.3–85.5)	52.4% (47.1–57.6)	96.4% (95–97.6)	0.927 (0.894–0.96)	68/1046/100/13	84% (74.1–91.2)	91.3% (89.5–92.8)	90.8% (89–92.3)	40.5% (33–48.3)	98.8% (97.9–99.3)
**KMA model**	0.723 (0.693–0.753)	129/108/1308/106	54.9% (61.4)	7.6% (6.3–9.148.3-)	14.4% (12.7–16.1)	9% (7.5–10.6)	50.5% (43.6–57.4)	0.728 (0.678–0.778)	43/1395/171/42	50.6% (39.5–61.6)	89.1% (87.4–90.6)	87.1% (85.4–88.7)	20.1% (14.9–26.1)	97.1% (96.1–97.9)
**MEWS**	0.598 (0.563–0.633)	129/314/1023/98	56.8% (50.1–63.4)	23.5% (21.2–25.9)	28.3% (26.1–30.6)	11.2% (9.4–13.2)	76.2% (71.8–80.2)	0.631 (0.574–0.689)	41/1112/371/40	50.6% (39.3–61.9)	75% (72.7–77.2)	73.7% (71.5–75.9)	10% (7.2–13.3)	96.5% (95.3–97.5)

The results of other machine learning algorithms can be found in Supplementary Table S5. Values in parentheses are 95% confidence intervals. *OLR* ordinal logistic regression, *AUC* area under the receiver operator characteristics curve, *TP* true positive, *TN* true negative, *FP* false positive, *FN* false negative, *NPV* negative predictive value, *KMA* Korean Medical Association, *MEWS* modified Early Warning Score.

*The use of a ventilator or extracorporeal membrane oxygenation machine.

#### Development and internal validation of machine learning model

Machine learning models showed better performances than the conventional protocols (Table 2 and Supplementary Table S5). In the internal validation, with the OLR algorithm, the AUCs of Models 1, 2A, 2B, 3, and 4 were 0.880 (95% CI, 0.855–0.904), 0.889 (0.865–0.912), 0.866 (0.841–0.892), 0.894 (0.871–0.917), and 0.907 (0.884–0.929) in predicting whether a patient will require at least oxygen therapy, and 0.903 (0.869–0.937), 0.905 (0.869–0.940), 0.922 (0.892–0.953), and 0.927 (0.894–0.960) in predicting whether a patient will need critical care or die, respectively (Table 2). The other machine learning algorithms—random forest (RF), linear support vector machine (L-SVM), and SVM with a radial basis function kernel (R-SVC)—did not show superior performances to the OLR model (Supplementary Table S5).

**Figure 2:**
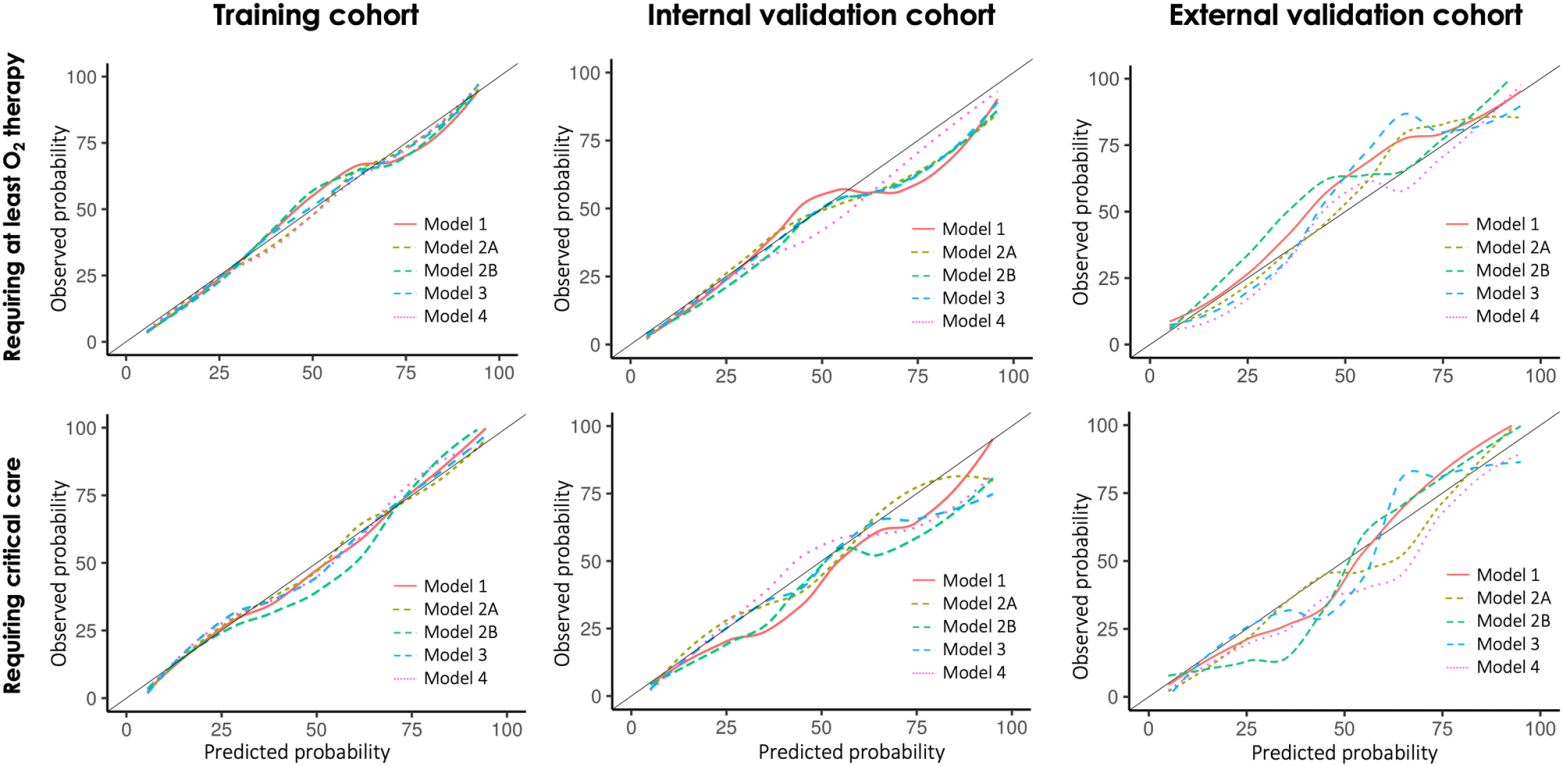
Calibration plot.

**Figure 3:**
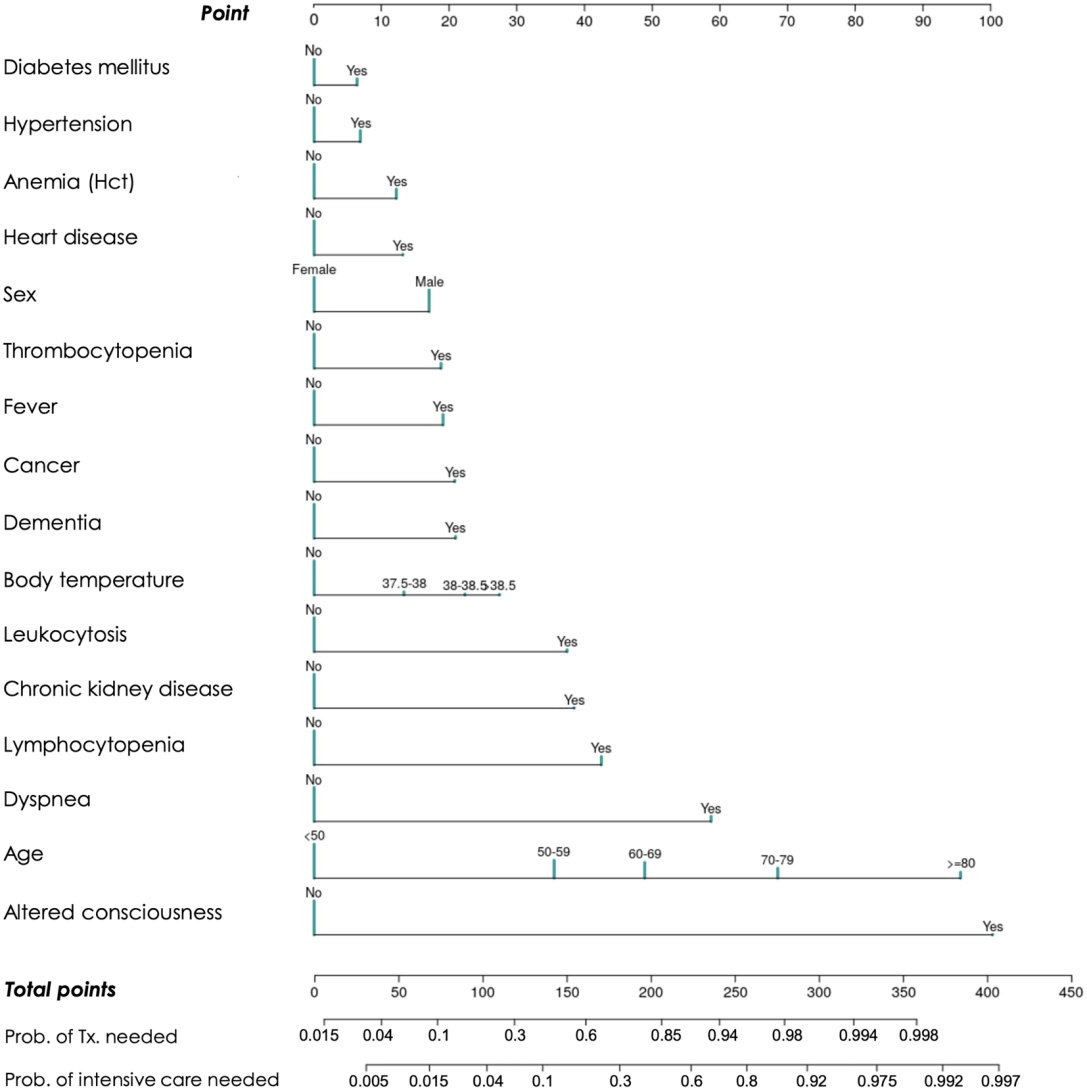
Nomogram of ordinal logistic regression model using all the predictors (Model 4). The nomogram is used by first giving each variable a score on the ‘Point’ scale. The points for all variables are then added to obtain the total points and a vertical line is drawn from the ‘Total points’ row to estimate the probability of requiring treatment and that of requiring critical care or death. The nomograms of the other models can be found in Supplementary Fig. S1.

The sensitivity, specificity, accuracy, precision, and negative predictive value (NPV) at different cutoff probabilities for the OLR models are presented in Supplementary Table S6. The models showed good calibration in the training and testing, especially in the probability range of < 50% (Fig. 2). Figure 3 shows the nomogram of OLR Model 4 to predict the probability of recovering without particular treatment and the probability of requiring critical care or death from COVID-19 (see Supplementary Fig. S1 for the nomograms of all the five models).

#### External validation

In the external validation, the AUCs of Models 1, 2A, 2B, 3, and 4 were 0.829 (95% CI, 0.786–0.869), 0.850 (0.809–0.895), 0.838 (0.796–0.879), 0.861 (0.822–0.902), and 0.871 (0.834–0.910) in predicting whether a patient will require at least oxygen therapy, and 0.827 (0.754–0.901), 0.833 (0.759–0.907), 0.833 (0.759–0.907), 0.851 (0.786–0.912), and 0.864 (0.802–0.916) in predicting whether a patient will need critical care or die, respectively (Table 3). The sensitivity, specificity, accuracy, precision, and NPV at the optimal cutoff probability for each model are presented in Table 3, and those at different cutoff probabilities can be interactively viewed on our website (https://nhimc.shinyapps.io/ih-psc/), where the results of validation ongoing in our institution are periodically updated; the results on the website will be different from those in this study after updates.

**Table 3:** Model performance in early prediction of prognosis in COVID19 patients in the external validation cohort.

Model	No significant treatment versus O_2_ therapy or more								No critical care required versus critical care* or death							
	AUC	Cutoff (%)	TP/TN/FP/FN	Sensitivity	Specificity	Accuracy	Precision	NPV	AUC	Cutoff (%)	TP/TN/FP/FN	Sensitivity	Specificity	Accuracy	Precision	NPV
1	0.829 (0.786–0.869)	30	82/260/46/38	68.3% (59.2–76.5)	85% (80.5–88.8)	80.3% (76.2–84)	64.1% (55.1–72.3)	87.2% (82.9–90.8)	0.827 (0.754–0.901)	11	29/304/84/9	76.3% (59.8–88.6)	78.4% (73.9–82.3)	78.2% (73.9–82)	25.7% (17.9–34.7)	97.1% (94.6–98.7)
2A	0.850 (0.809–0.895)	30	93/245/61/27	77.5% (69–84.6)	80.1% (75.1–84.4)	79.3% (75.2–83.1)	60.4% (52.2–68.2)	90.1% (85.9–93.4)	0.833 (0.759–0.907)	23	24/350/38/14	63.2% (46–78.2)	90.2% (86.8–93)	87.8% (84.3–90.7)	38.7% (26.6–51.9)	96.2% (93.6–97.9)
2B	0.838 (0.796–0.879)	20	93/235/71/27	77.5% (69–84.6)	76.8% (71.7–81.4)	77% (72.7–80.9)	56.7% (48.8–64.4)	89.7% (85.4–93.1)	0.833 (0.759–0.907)	60	24/350/38/14	63.2% (46–78.2)	90.2% (86.8–93)	87.8% (84.3–90.7)	38.7% (26.6–51.9)	96.2% (93.6–97.9)
3	0.861 (0.822–0.902)	29	92/250/56/28	76.7% (68.1–83.9)	81.7% (76.9–85.9)	80.3% (76.2–84)	62.2% (53.8–70.0)	89.9% (85.8–93.2)	0.851 (0.786–0.912)	14	26/333/55/12	68.4% (51.3–82.5)	85.8% (82–89.1)	84.3% (80.5–87.6)	32.1% (22.2–43.4)	96.5% (94–98.2)
4	0.871 (0.834–0.910)	39	95/254/46/25	79.2% (70.8–86.0)	84.7% (80.1–88.6)	83.1% (79.2–86.6)	67.4% (59.0–75.0)	91% (87.1–94.1)	0.864 (0.802–0.916)	12	31/290/92/7	81.6% (65.7–92.3)	75.9% (71.3–80.1)	76.4% (72.1–80.4)	25.2% (17.8–33.8)	97.6% (95.2–99)

Values in parentheses are 95% confidence intervals. *AUC* area under the receiver operator characteristics curve, *TP* true positive, *TN* true negative, *FP* false positive, *FN* false negative, *NPV* negative predictive value.

*The use of a ventilator or extracorporeal membrane oxygenation machine.

## Discussion

Our results demonstrate that a data-driven model to predict prognosis can be a good tool for early triage of COVID-19 patients. A significant shortcoming of the triage protocols that are not based on data is that risk factors are not weighted appropriately based on their effects on the outcome. For example, the WHO algorithm for COVID-19 triage and referral regards age > 60 years and the presence of relevant symptoms or co-morbidities as risk factors, but it does not put different weights on them^2^. However, if not treated as a continuous variable, age should be divided into multiple categories with appropriate weights because the risk continues to increase with age even after 60 years. Different symptoms or co-morbidities must also be weighted according to their importance when assessing the patients’ status for triage. For example, in the current study, subjective fever, dyspnea, and altered consciousness were independent risk factors for severe illness, while other symptoms such as cough, sputum production, sore throat, myalgia, and diarrhea were not.

Our final prediction model used the OLR algorithm. We chose the OLR over the other machine learning algorithms (i.e., RF, L-SVM, and R-SVM) because it showed comparable performances to the other algorithms in the final evaluation. Furthermore, a linear model like the OLR is more interpretable and easier to use even without a computer device, as nomograms can be used instead. We also observed the linear model’s superiority in predicting COVID-19 prognosis in our previous study in which we developed a model to predict the risk of COVID-19 mortality based on demographics and medical claim data^15^.

Our current model has a few differences compared to other proposed models. Above all, our main purpose was to develop an easy-to-use prediction model that can be used widely in various real-world fields. This was another reason that we preferred a linear prediction model to other complex machine learning algorithms; simply by knowing the coefficients of the linear model, anyone can calculate the predicted risk using various methods: the nomogram or web-based application we developed, or even paper-and-pencil calculation. Several published prognostic models have also used linear logistic regression and proposed nomograms possibly for the same reason^10,13,16,17,20^. However, those models were designed to be used for hospitalized patients, requiring information that is usually obtained after hospitalization such as laboratory test results or imaging studies. In contrast, our model is intended to be used in various situations, not only for hospitalized patients but also for early triage immediately after the diagnosis. Therefore, our model uses different algorithms depending on the available variable subsets. Health workers sometimes need to triage newly diagnosed COVID-19 patients even by a phone call alone, and patients commonly do not know their underlying disease exactly. Therefore, we expect that our model’s flexibility may make our model distinct from previous models and lead to a more widespread use. Lastly, we divided disease severity into three categories. This is more helpful than the binary categorization (i.e., recovery vs. mortality), because not all medical facilities capable of oxygen therapy can also provide critical care, such as mechanical ventilation or ECMO.

The predictors chosen in this study are not much different from the known risk factors of developing into critical conditions from COVID-19^21^. However, it was unexpected that chronic obstructive pulmonary disease (COPD), a known strong risk factor, was not selected as a predictor. We assume that this is because there were only 40 patients with COPD in the entire cohort, of whom 65% had dyspnea, and the disease severity of COPD might have varied widely. Thus, it is likely that the number of COPD cases was too small (became even smaller after the training-validation set split) to play a significant role independently from the other strong predictors.

There are limitations to our current model. First, since we trained and validated our model on Koreans’ data, it is unsure whether it can be generalizable to patient cohorts in other countries or races. We hope to be able to develop a triage model that can be used globally through collaboration. Second, we converted continuous variables such as blood test results into categorical variables, which may have resulted in some loss of information. Our intention was, however, to prevent small differences in continuous variables (which could be more of a noise than a true signal in terms of prediction) from overfitting models. Furthermore, all the variables categorized in this study have well-established cutoff values classifying them into categories (e.g., normal vs, abnormal). Third, our data lacked some important variables, such as smoking, respiratory rate, and oxygen saturation, and had missing values in some of the Tiers-2/3/4 variables, which may have affected the training and performance of the algorithms using those variables. We did not perform imputation for missing values because we did not want the uncertainty and potential bias from imputation, and imputation for missing values did not make significant differences in our preliminary analysis. Lastly, we did not experiment more machine learning algorithms such as extreme gradient boost. Thus, we cannot conclude that OLR is superior to all other machine learning algorithms.

In conclusion, we developed and validated a set of models that can be used for disease severity prediction and triage or referral of COVID-19 patients. Our prediction model showed a good performance with age, sex, symptoms, and the information on underlying disease used as predictors. The model performance was enhanced when further information on vital signs and blood test results were also used.

## Materials and methods

### Ethical approval

The Institutional Review Board of National Health Insurance Service Ilsan Hospital (NHIMC 2020-08-018 and 2021-02-023) approved this retrospective Health Insurance Portability and Accountability Act-compliant cohort study and waived the informed consent from the participants. We performed all methods in accordance with relevant guidelines and regulations.

### Data source and patients

This study used two datasets. For model development and internal validation, we used a dataset containing the epidemiologic and clinical information of patients diagnosed with COVID-19 in South Korea, which the Korea Disease Control and Prevention Agency collected, anonymized, and provided to researchers for the public interest. The data included 5,628 patients who either were cured or died from COVID-19 infection by April 30, 2020. After excluding 32 patients who lacked the information on disease severity or the presence or absence of symptoms, a total of 5,596 patients comprised the model development cohort. The dataset was randomly divided into training and internal validation cohorts with a ratio of 7:3 while preserving the disease severity distribution. We trained and optimized models using the training cohort and validation them on the internal validation cohort.

**Figure 4:**
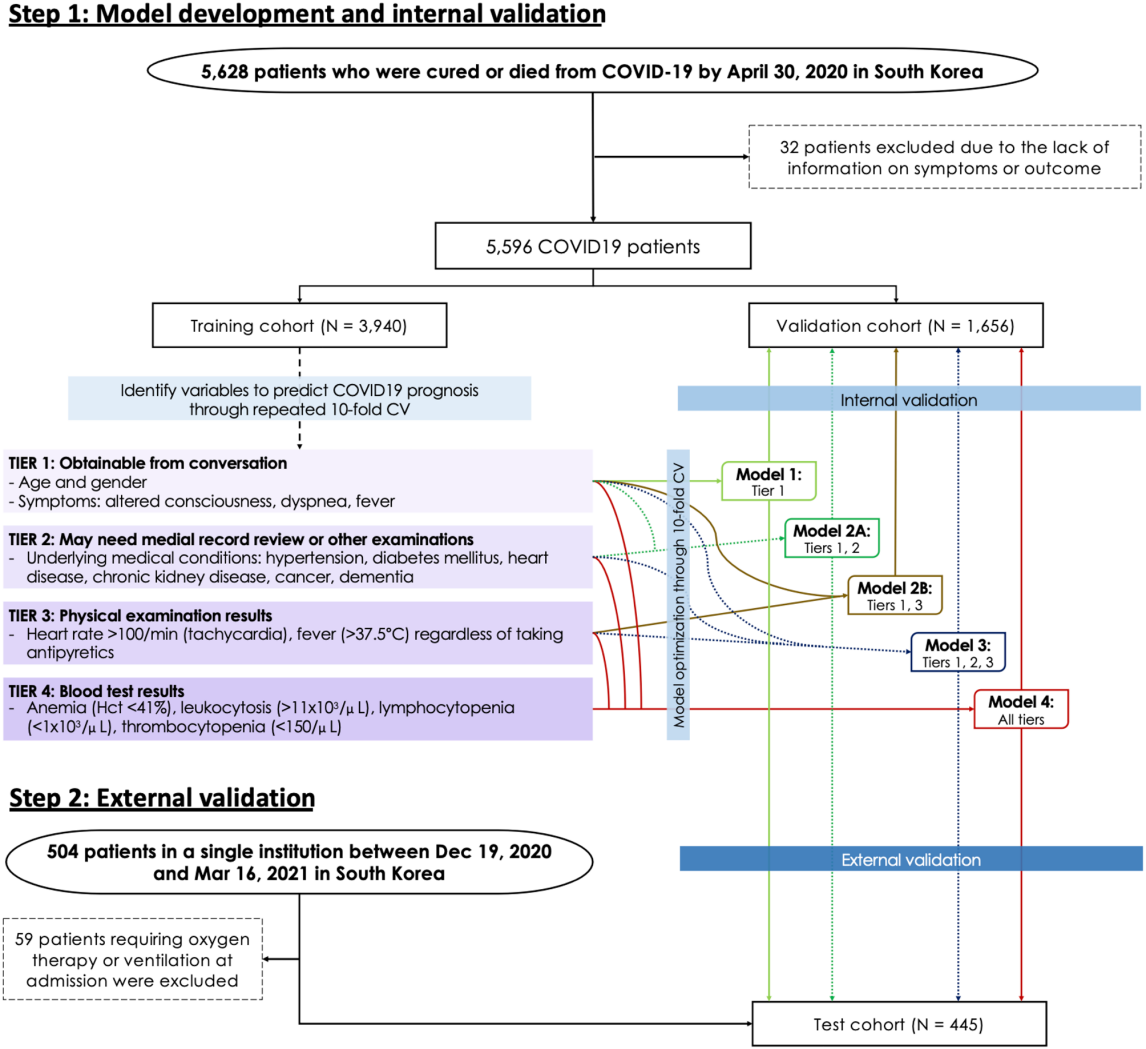
Study flow.

For external validation, we used a cohort of COVID-19 patients treated in National Health Insurance Service, Korea, between December 19, 2020 and March 16, 2021. After excluding 59 patients who were referred with severe conditions requiring oxygen therapy or mechanical ventilation at the time of admission, a total of 445 patients comprised this external validation cohort (Fig. 4).

The outcome variable was the worst severity during the disease course, determined by the type of treatment required: (1) none or supportive treatment, (2) oxygen therapy, (3) critical care such as mechanical ventilation or ECMO, or death from COVID-19 infection.

### Variables in four different tiers based on accessibility

We intended to develop a model that can be used flexibly in real-world circumstances where some of the variables may not be available. Therefore, we categorized variables into four tiers based on their accessibility (Table 1 and Fig. 4).

#### Tier 1: basic demographics and symptoms

Tier 1 variables can be obtained by simply asking a patient questions: age, sex, body mass index, pregnancy, and symptoms. The symptoms included were subjective fever, cough, sputum, dyspnea, altered consciousness, headache, rhinorrhea, myalgia, sore throat, fatigue, nausea or vomiting, and diarrhea. We separated this group of variables from others because there could be times when we need to triage a patient quickly without physical contact.

#### Tier 2: underlying diseases

Tier 2 variables are underlying medical conditions: hypertension, DM, heart disease, asthma, COPD, CKD, chronic liver disease, cancer, autoimmune disease, and dementia. We categorized these variables into a separate group because sometimes patients may not know exactly their underlying medical conditions. In this case, further actions may be required, including reviewing medical records or other examinations.

#### Tier 3: vital signs

Tier 3 variables are blood pressure, body temperature, and heart rate. Our data lacked information on breathing rate. We separated these variables from the first two tiers because these can be obtained only when a patient visits a medical facility or can measure their vital signs on their own. Blood pressure and heart rate were transformed into binary categorical variables by merging categories that were not significantly associated with disease severity based on the preliminary results in the training cohort: severe hypertension (systolic blood pressure ≥ 160 mmHg) and tachycardia (heart rate ≥ 100 bpm). We assumed that many patients had their body temperature measured while taking antipyretics, although our data did not contain the information on such patients’ proportion.

#### Tier 4: Blood test results

Tier 4 variables are hemoglobin, hematocrit, white blood cell (WBC) count, lymphocyte count, and platelet count, which are available only after a blood test. As with Tier 3, these variables were also transformed into binary categorical variables: anemia (hematocrit < 40%), leukocytosis (WBC ≥ 11 × 10^3^/µL), lymphocytopenia (lymphocyte < 1,000/µL), and thrombocytopenia (platelet < 150,000/µL).

### Predictor selection

To identify robust and stable predictors, we repeated tenfold cross-validation (CV) 100 times with shuffling and choose variables that were selected more than 900 times out of 1,000 trials (> 90%) based on two algorithms: Least Absolute Selection and Shrinkage Operator (LASSO) and RF. A variable was selected if its coefficient was non-zero on LASSO, and its variable importance on RF was positive^22,23^.

### Development of prediction models

We used four machine learning algorithms: OLR, multivariate RF, L-SVM, and R-SVM. For each algorithm, five models were created using one of the following five predictor sets: predictors chosen from the Tier 1 variables (Model 1), Tiers 1/2 variables (Model 2A), Tiers 1/3 variables (Model 2B), Tiers 1/2/3 variables (Model 3), and Tiers 1/2/3/4 variables (Model 4). We optimized the hyperparameters for RF and SVM through a tenfold CV with a grid search in the training cohort, using AUC as an evaluation metric.

OLR is a general term for logistic regression with (usually more than 2) ordinal outcomes. Among different OLR models, we used proportional odds model which assumes that the effects of input variables are proportional across the different outcomes, as interpretation under this model deemed logical and meaningful in our case. In case of the current study, as the outcome of each patient, denoted as *Y* here, is classified into one of three categories: supportive treatment (*y*_1_), oxygen therapy (*y*_2_), and critical care or death (*y*_3_), the dependency of *Y* on *X* (a vector of input variables of *x*_1_, *x*_2_, _…_, *x*_p_) can be expressed as:

$$ log\left[ {\frac{{Pr(Y \ge y_{j} |X)}}{{1 - Pr(Y \ge y_{j} |X)}}} \right] = \alpha_{j} + \mathop \sum \limits_{i = 1}^{p} x_{i} \beta_{i} \left( {i = 1, 2, \ldots , p; j = 2, 3} \right) $$

where Pr(*Y* ≥ *y*_j_) is the cumulative probability of the outcome; *α*_j_ is a respective intercept; and *β*_i_ is a coefficient corresponding to the *x*_i_ variable. Readers interested in more detailed explanation are referred to the paper by Singh et al.^24^.

In this study, the number of outcomes were more than two (not binary), which is considered multiclass or multinomial classification in machine learning. OLR and RF can perform multiclass classification inherently. With SVM, we performed multiclass classification using the one-vs.-rest scheme^25^.

### Validation of prediction models in comparison with current protocols

We validated the optimized models in the internal validation cohort after fitting them onto the entire training dataset. Based on the probabilities for each outcome category, we assessed the diagnostic performance of each model for whether or not a patient will require treatment (Outcome 1 vs. 2/3), and whether or not a patient will require critical care or die (Outcome 1/2 vs. 3). Sensitivity, specificity, accuracy, precision, and NPV according to different probability cutoffs were calculated, in addition to AUC. We also drew calibration curves to compare the predicted and observed probabilities visually.

As a baseline for comparison, we also tested two protocols used to triage a newly diagnosed COVID-19 patient: a protocol proposed by the KMA and MEWS^5,26^. These are two of the protocols that the Korean government currently recommends using with some modifications depending on the situation^5^. Since we did not have information on smoking status, oxygen saturation, and respiratory rate, these variables were considered normal when applying the protocols. These protocols are described in detail in Supplementary Tables S7 and S8.

We tested the final model in the external validation cohort in the same manner as the internal validation. We also developed a web-based application for calculating the probability of requiring oxygen therapy or critical care based on the results of this study, where users also can view the ongoing validation results in our institution (https://nhimc.shinyapps.io/ih-psc/).

## Supplementary Information

Supplementary Information.
