# Supplementary material for: Development and validation of a prognostic model for early triage of patients diagnosed with COVID-19
Source: Sci Rep. 2021 Nov 9;11:21923. doi: 10.1038/s41598-021-01452-7 (PMC8578640; doi:10.1038/s41598-021-01452-7)
Supplement: Supplementary file 1 — Supplementary Information. [file 41598_2021_1452_MOESM1_ESM.docx]

**Supplementary Materials**

Supplementary materials for “Development and validation of a prognostic model for early triage of patients diagnosed with COVID-19”

Title: Development and validation of a prognostic model for early triage of patients diagnosed with COVID-19

Chansik An^1,2^, Hyun Cheol Oh^3*^, Jung Hyun Chang^4^, Seung-Jin Oh^5^, Jung Mo Lee^6^, Chang Hoon Han^6^, and Seong Woo Kim^7^

^1^Research Institute, National Health Insurance Service Ilsan Hospital, Goyang, Korea

^2^Department of Radiology, National Health Insurance Service Ilsan Hospital, Goyang, Korea

^3^Department of Orthopedic Surgery, National Health Insurance Service Ilsan Hospital, Goyang, Korea

^4^Department of Otolaryngology-Head and Neck Surgery, National Health Insurance Service Ilsan Hospital, Goyang, Korea

^5^Division of Cardiology, Department of Internal Medicine, National Health Insurance Service Ilsan Hospital, Goyang, Korea

^6^Division of Pulmonology, Department of Internal Medicine, National Health Insurance Service Ilsan Hospital, Goyang, Korea

^7^Department of Physical Medicine and Rehabilitation, National Health Insurance Service Ilsan Hospital, Goyang, Korea

***Corresponding author:**

Hyun Cheol Oh, MD

Department of Orthopedic Surgery, National Health Insurance Service Ilsan Hospital, Goyang, Korea.

Phone: +82-31-900-6956; E-mail: [hyuncoh@nhimc.or.kr](mailto:hyuncoh@nhimc.or.kr)

**Table of contents**

Supplementary Figure S1: OLR nomograms

Supplementary Table S1. Characteristics of patients in the training and internal validation subcohorts of the model development cohort

Supplementary Table S2. Characteristics of patients in the external validation cohort

Supplementary Table S3. The full results of predictor selection

Supplementary Table S4. Odds ratio of predictors for COVID-19 severity by multivariable ordinal logistic regression in the entire dataset

Supplementary Table S5. The full results of model performances

Supplementary Table S6. Model performances by cutoff probabilities in the internal validation cohort

Supplementary Table S7: Criteria for COVID-19 severity proposed by the Korea Medical Association (KMA)

Supplementary Table S8. Modified Early Warning Score (MEWS)

**Supplementary Figure S1. Ordinal logistic regression nomograms of Models 1, 2A, 2B, 3, and 4.** The nomogram is used by first giving each variable a score on the ‘Point’ scale. The points for all variables are then added to obtain the total points and a vertical line is drawn from the ‘Total points’ row to estimate the probability of requiring treatment and that of requiring critical care or death.

(A) Ordinal logistic regression nomograms of Model 1


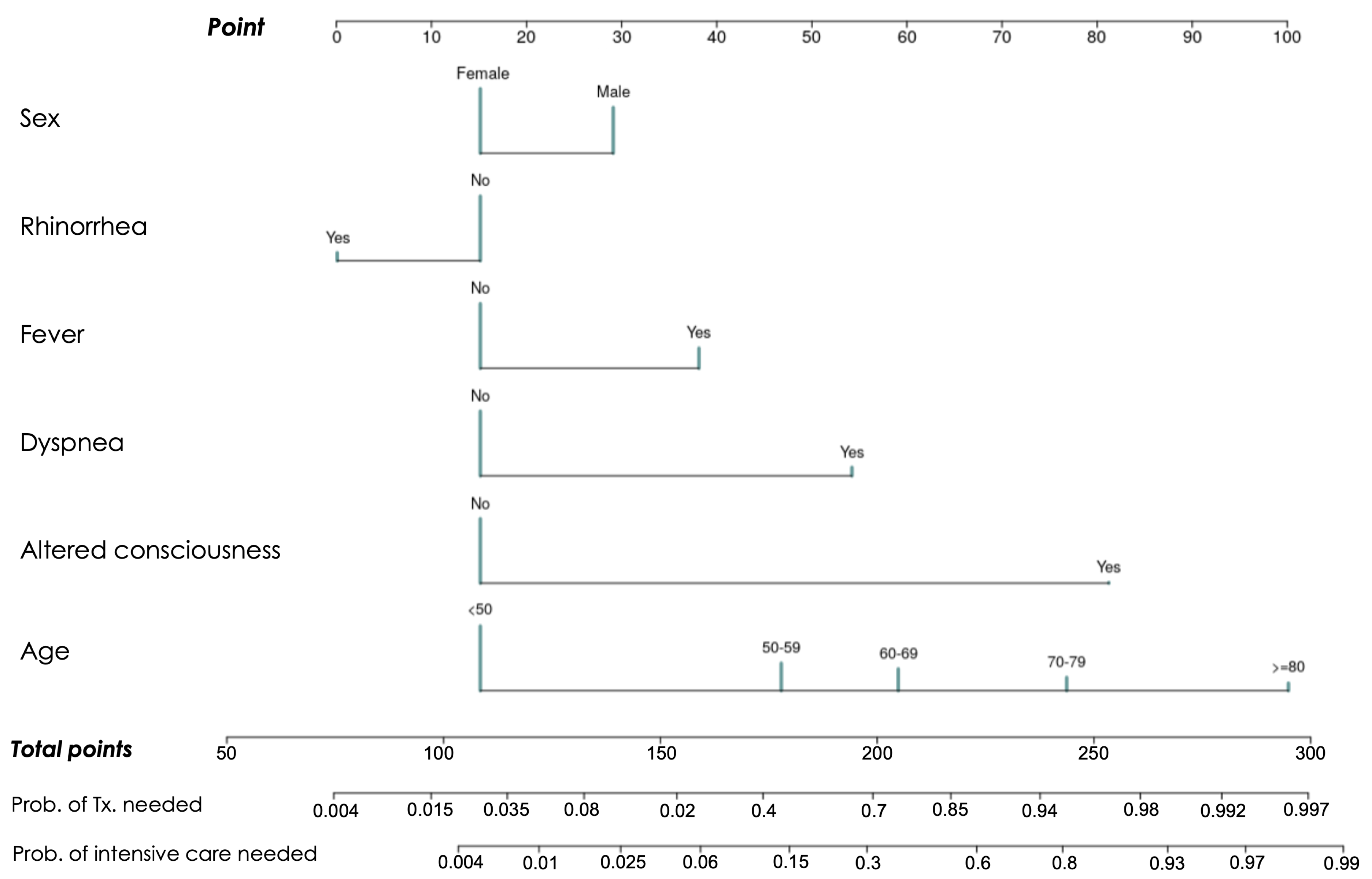


(B) Ordinal logistic regression nomograms of Model 2A


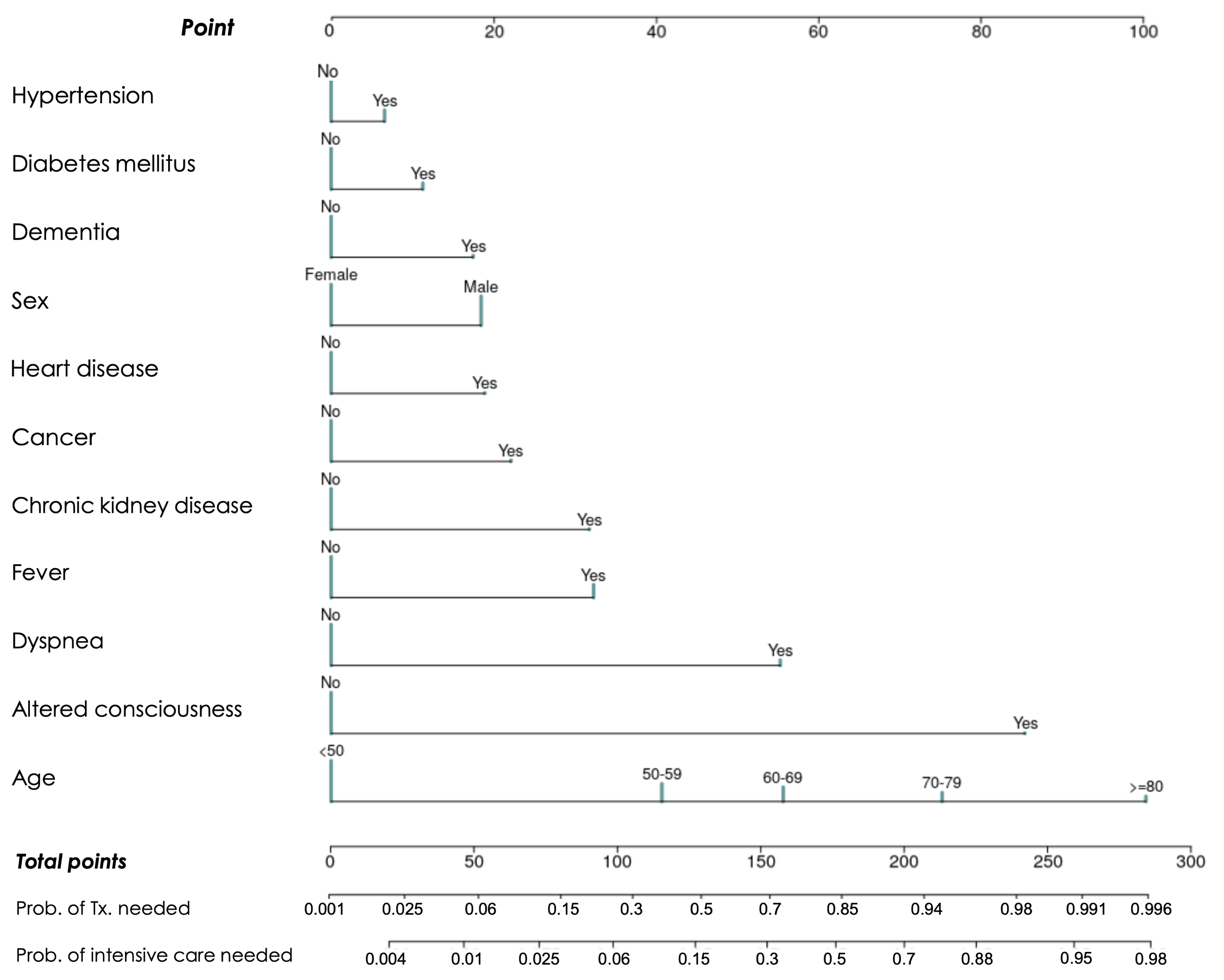


(C) Ordinal logistic regression nomograms of Model 2B


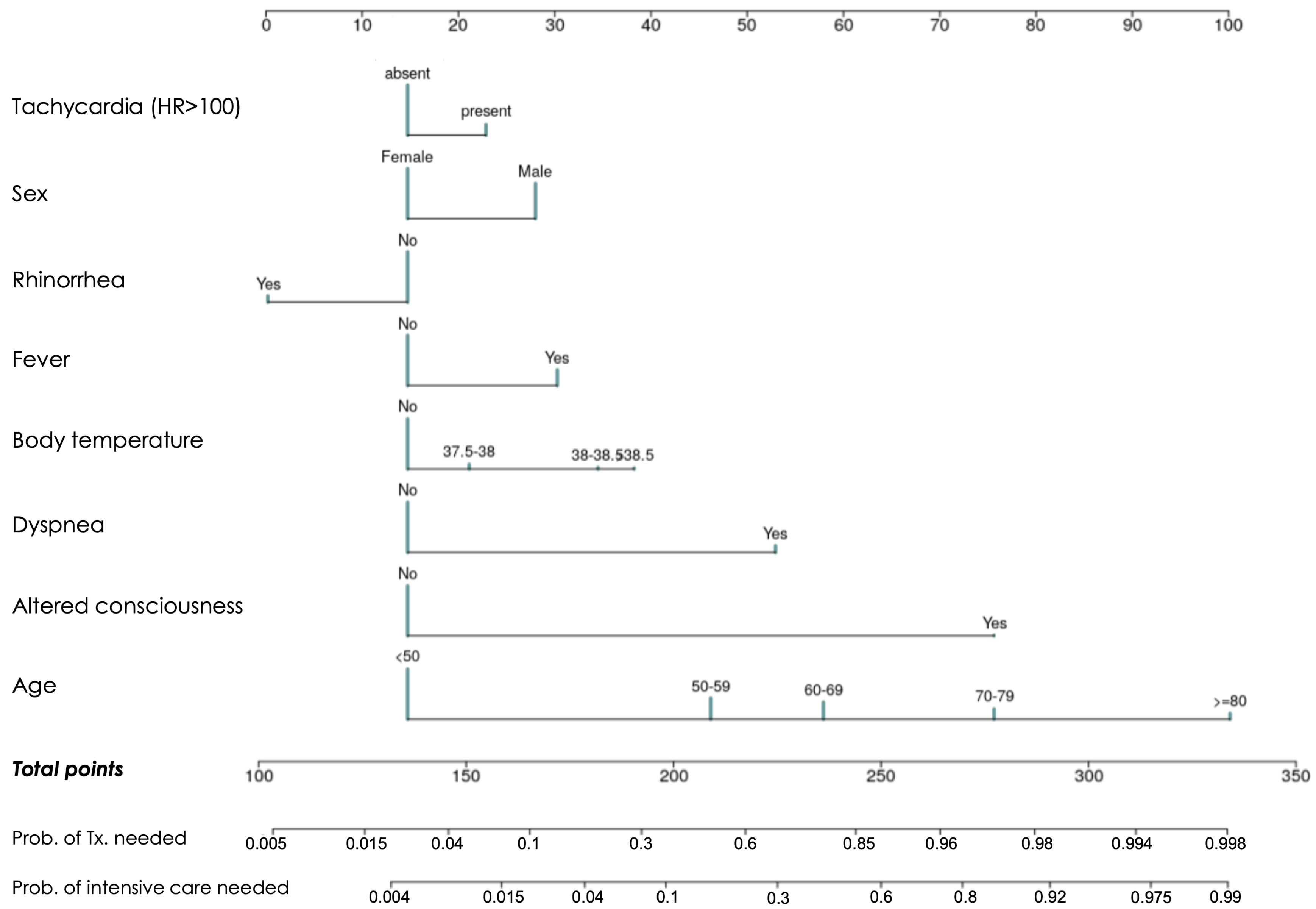


(D) Ordinal logistic regression nomograms of Model 3


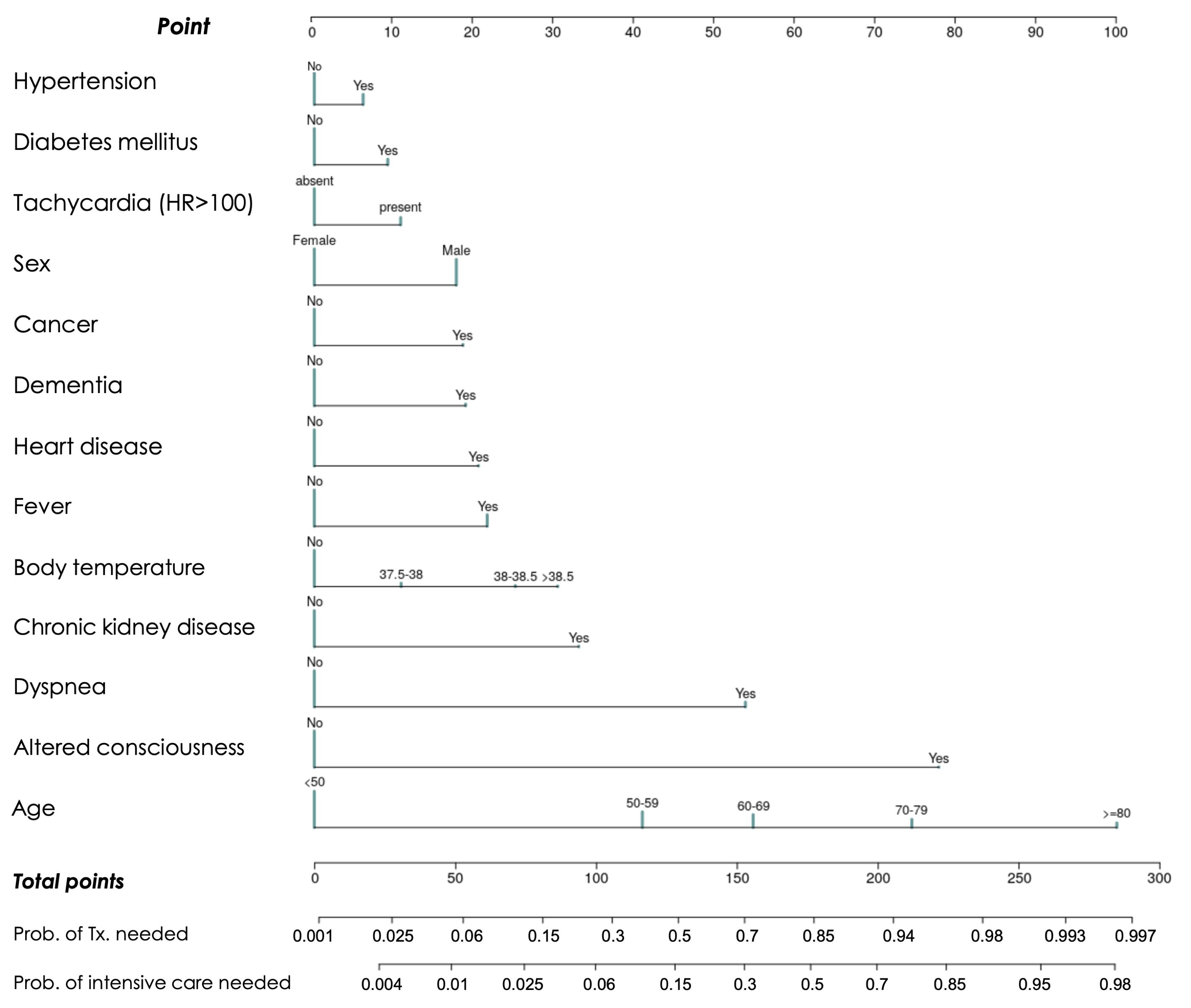


(E) Ordinal logistic regression nomograms of Model 4


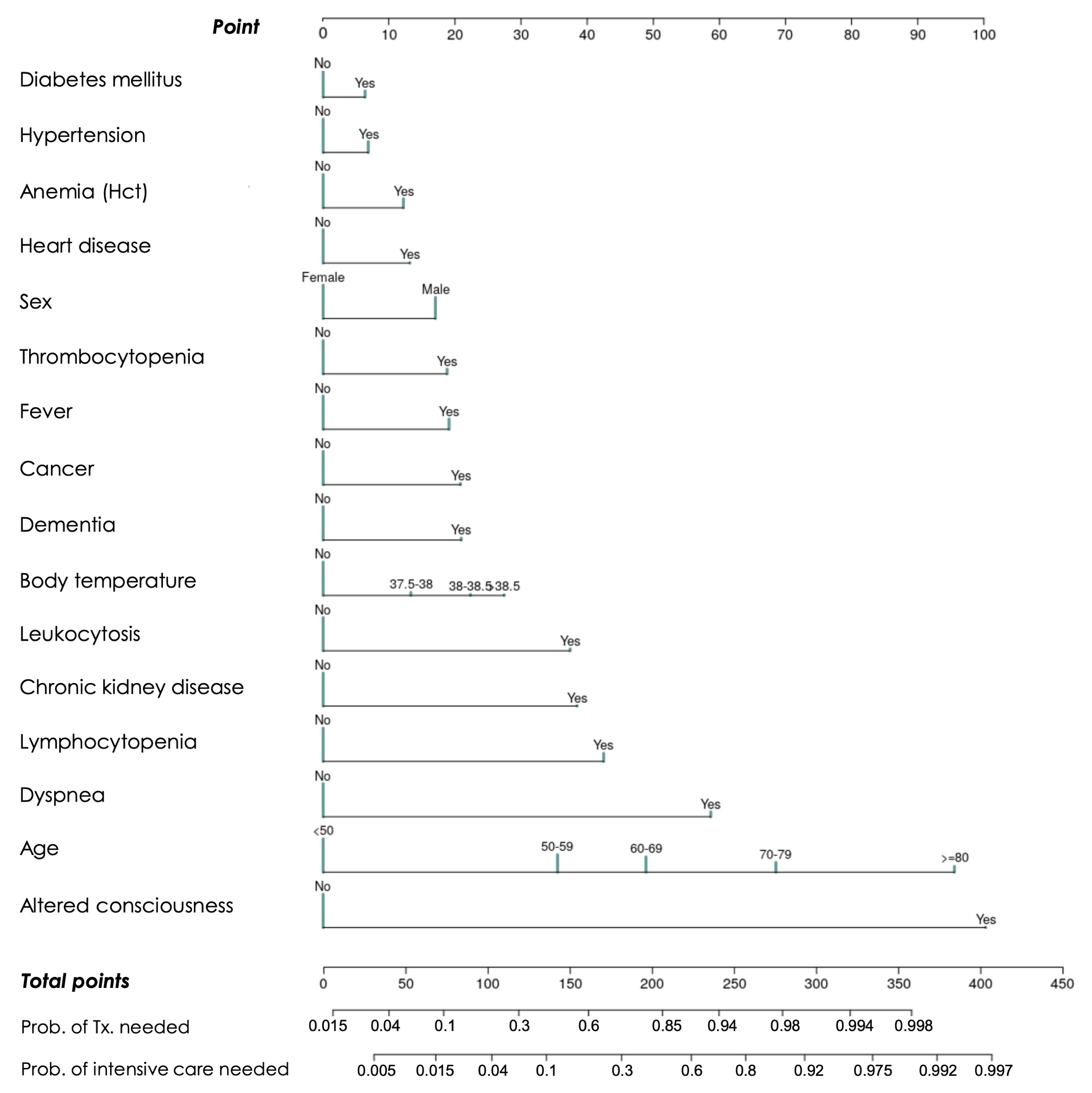


**Supplementary Table S1.** Characteristics of patients in the training and internal validation subcohorts of the model development cohort

| **Variable** | | **Training cohort**  **(n = 3940)** | **Test cohort**  **(n = 1656)** | ***p-*value** | **Total**  **(n = 5596)** |
| --- | --- | --- | --- | --- | --- |
| Age | 0-9 years | 43 (65.2%) | 23 (34.8%) | 0.920 | 66 (100%) |
|  | 10-19 years | 148 (72.2%) | 57 (27.8%) |  | 205 (100%) |
|  | 20-29 years | 774 (69.8%) | 335 (30.2%) |  | 1109 (100%) |
|  | 30-39 years | 400 (71.2%) | 162 (28.8%) |  | 562 (100%) |
|  | 40-49 years | 522 (70.6%) | 217 (29.4%) |  | 739 (100%) |
|  | 50-59 years | 801 (70.3%) | 339 (29.7%) |  | 1140 (100%) |
|  | 60-69 years | 633 (69.9%) | 273 (30.1%) |  | 906 (100%) |
|  | 70-79 years | 383 (70.3%) | 162 (29.7%) |  | 545 (100%) |
|  | ≥80 years | 217 (67%) | 107 (33%) |  | 324 (100%) |
| Sex | Female | 2297 (69.8%) | 992 (30.2%) | 0.677 | 3289 (100%) |
|  | Male | 1624 (70.4%) | 683 (29.6%) |  | 2307 (100%) |
| Pregnancy | No | 3899 (70%) | 1669 (30%) | 0.923 | 5568 (100%) |
|  | Yes | 14 (73.7%) | 5 (26.3%) |  | 19 (100%) |
|  | Missing | 8 (88.9%) | 1 (11.1%) |  | 9 (100%) |
| Body mass index (kg/cm^2^) | <18.5 | 177 (68.3%) | 82 (31.7%) | 0.294 | 259 (100%) |
|  | 18.5-23 | 1271 (68.6%) | 583 (31.4%) |  | 1854 (100%) |
|  | 23-25 | 735 (71.2%) | 298 (28.8%) |  | 1033 (100%) |
|  | 25-30 | 752 (72%) | 293 (28%) |  | 1045 (100%) |
|  | >30 | 142 (68.6%) | 65 (31.4%) |  | 207 (100%) |
|  | Missing | 844 (70.5%) | 354 (29.5%) |  | 1198 (100%) |
| Subjective fever | Absent | 2992 (69.7%) | 1302 (30.3%) | 0.263 | 4294 (100%) |
|  | Present | 929 (71.4%) | 373 (28.6%) |  | 1302 (100%) |
| Cough | Absent | 2288 (70.1%) | 977 (29.9%) | 1.000 | 3265 (100%) |
|  | Present | 1633 (70.1%) | 698 (29.9%) |  | 2331 (100%) |
| Sputum | Absent | 2810 (70.5%) | 1175 (29.5%) | 0.265 | 3985 (100%) |
|  | Present | 1111 (69%) | 500 (31%) |  | 1611 (100%) |
| Dyspnea | Absent | 3471 (70.4%) | 1460 (29.6%) | 0.163 | 4931 (100%) |
|  | Present | 450 (67.7%) | 215 (32.3%) |  | 665 (100%) |
| Sore throat | Absent | 3312 (70.1%) | 1412 (29.9%) | 0.904 | 4724 (100%) |
|  | Present | 609 (69.8%) | 263 (30.2%) |  | 872 (100%) |
| Rhinorrhea | Absent | 3493 (70.2%) | 1486 (29.8%) | 0.722 | 4979 (100%) |
|  | Present | 428 (69.4%) | 189 (30.6%) |  | 617 (100%) |
| Myalgia | Absent | 3275 (70%) | 1402 (30%) | 0.901 | 4677 (100%) |
|  | Present | 646 (70.3%) | 273 (29.7%) |  | 919 (100%) |
| Fatigue | Absent | 3759 (70.1%) | 1604 (29.9%) | 0.912 | 5363 (100%) |
|  | Present | 162 (69.5%) | 71 (30.5%) |  | 233 (100%) |
| Headache | Absent | 3251 (70.2%) | 1382 (29.8%) | 0.742 | 4633 (100%) |
|  | Present | 670 (69.6%) | 293 (30.4%) |  | 963 (100%) |
| Nausea or vomiting | Absent | 3745 (70%) | 1607 (30%) | 0.517 | 5352 (100%) |
|  | Present | 176 (72.1%) | 68 (27.9%) |  | 244 (100%) |
| Diarrhea | Absent | 3559 (70.1%) | 1521 (29.9%) | 1.000 | 5080 (100%) |
|  | Present | 362 (70.2%) | 154 (29.8%) |  | 516 (100%) |
| Altered consciousness | Absent | 3894 (70%) | 1667 (30%) | 0.464 | 5561 (100%) |
|  | Present | 27 (77.1%) | 8 (22.9%) |  | 35 (100%) |
| Diabetes mellitus | Absent | 3432 (69.9%) | 1477 (30.1%) | 0.526 | 4909 (100%) |
|  | Present | 489 (71.2%) | 198 (28.8%) |  | 687 (100%) |
| Hypertension | Absent | 3098 (70.4%) | 1300 (29.6%) | 0.257 | 4398 (100%) |
|  | Present | 823 (68.7%) | 375 (31.3%) |  | 1198 (100%) |
| Heart disease | Absent | 3878 (70%) | 1659 (30%) | 0.740 | 5537 (100%) |
|  | Present | 43 (72.9%) | 16 (27.1%) |  | 59 (100%) |
| Asthma | Absent | 3833 (70.1%) | 1635 (29.9%) | 0.817 | 5468 (100%) |
|  | Present | 88 (68.8%) | 40 (31.2%) |  | 128 (100%) |
| Chronic obstructive pulmonary disease | Absent | 3894 (70.1%) | 1662 (29.9%) | 0.855 | 5556 (100%) |
|  | Present | 27 (67.5%) | 13 (32.5%) |  | 40 (100%) |
| Chronic kidney disease | Absent | 3882 (70.1%) | 1659 (29.9%) | 1.000 | 5541 (100%) |
|  | Present | 39 (70.9%) | 16 (29.1%) |  | 55 (100%) |
| Cancer | Absent | 3819 (70.1%) | 1632 (29.9%) | 1.000 | 5451 (100%) |
|  | Present | 102 (70.3%) | 43 (29.7%) |  | 145 (100%) |
| Chronic liver disease | Absent | 3638 (70.1%) | 1552 (29.9%) | 0.755 | 5190 (100%) |
|  | Present | 60 (72.3%) | 23 (27.7%) |  | 83 (100%) |
|  | missing | 223 (69%) | 100 (31%) |  | 323 (100%) |
| Autoimmune disease | Absent | 3669 (70.2%) | 1560 (29.8%) | 0.446 | 5229 (100%) |
|  | Present | 24 (63.2%) | 14 (36.8%) |  | 38 (100%) |
|  | missing | 228 (69.3%) | 101 (30.7%) |  | 329 (100%) |
| Dementia | Absent | 3538 (70.1%) | 1508 (29.9%) | 1.000 | 5046 (100%) |
|  | Present | 157 (70.1%) | 67 (29.9%) |  | 224 (100%) |
|  | missing | 226 (69.3%) | 100 (30.7%) |  | 326 (100%) |
| Heart rate (beat/min) | Bradycardia (<60) | 81 (75%) | 27 (25%) | 0.490 | 108 (100%) |
|  | Normal (60-100) | 3079 (69.9%) | 1324 (30.1%) |  | 4403 (100%) |
|  | Tachycardia (>100) | 666 (69.4%) | 293 (30.6%) |  | 959 (100%) |
|  | Missing | 95 (75.4%) | 31 (24.6%) |  | 126 (100%) |
| Body temperature (°C) | <37.5 | 3431 (70.2%) | 1454 (29.8%) | 0.785 | 4885 (100%) |
|  | 37.5-38 | 316 (68.3%) | 147 (31.7%) |  | 463 (100%) |
|  | 38-38.5 | 92 (67.6%) | 44 (32.4%) |  | 136 (100%) |
|  | 38.5≥38.5 | 53 (72.6%) | 20 (27.4%) |  | 73 (100%) |
|  | Missing | 29 (74.4%) | 10 (25.6%) |  | 39 (100%) |
| Systolic blood pressure (mmHg) | <120 | 928 (71.1%) | 378 (28.9%) | 0.196 | 1306 (100%) |
|  | 120-129 | 823 (72.3%) | 315 (27.7%) |  | 1138 (100%) |
|  | 130-139 | 745 (68.8%) | 338 (31.2%) |  | 1083 (100%) |
|  | 140-159 | 972 (68.6%) | 445 (31.4%) |  | 1417 (100%) |
|  | ≥160 | 353 (68.8%) | 160 (31.2%) |  | 513 (100%) |
|  | Missing | 100 (71.9%) | 39 (28.1%) |  | 139 (100%) |
| Diastolic blood pressure (mmHg) | <80 | 1472 (70%) | 630 (30%) | 0.597 | 2102 (100%) |
|  | 80-89 | 1265 (70.4%) | 531 (29.6%) |  | 1796 (100%) |
|  | 90-99 | 723 (68.5%) | 332 (31.5%) |  | 1055 (100%) |
|  | ≥100 | 361 (71.6%) | 143 (28.4%) |  | 504 (100%) |
|  | Missing | 100 (71.9%) | 39 (28.1%) |  | 139 (100%) |
| Hemoglobin (g/dL) | Anemia | 718 (70%) | 307 (30%) | 0.082 | 1025 (100%) |
|  | Normal^*^ | 1778 (70.5%) | 745 (29.5%) |  | 2523 (100%) |
|  | Elevated | 349 (65.6%) | 183 (34.4%) |  | 532 (100%) |
|  | Missing | 1076 (71%) | 440 (29%) |  | 1516 (100%) |
| Hematocrit (%) | Anemia | 613 (70.3%) | 259 (29.7%) | 0.230 | 872 (100%) |
|  | Normal^**^ | 1846 (70.2%) | 784 (29.8%) |  | 2630 (100%) |
|  | Elevated | 382 (66.7%) | 191 (33.3%) |  | 573 (100%) |
|  | Missing | 1080 (71%) | 441 (29%) |  | 1521 (100%) |
| White blood cell count (×10^3^/µL) | Leukocytopenia (<4) | 487 (70.7%) | 202 (29.3%) | 0.318 | 689 (100%) |
|  | Normal (4-11) | 2203 (69.8%) | 951 (30.2%) |  | 3154 (100%) |
|  | Leukocytosis (≥ 11) | 156 (65.5%) | 82 (34.5%) |  | 238 (100%) |
|  | Missing | 1075 (71%) | 440 (29%) |  | 1515 (100%) |
| Lymphocyte count (×10^3^/µL) | Lymphocytopenia (<1) | 546 (69.6%) | 239 (30.4%) | 0.319 | 785 (100%) |
|  | Normal (1-4.8) | 2258 (69.7%) | 980 (30.3%) |  | 3238 (100%) |
|  | Lymphocytosis (>4.8) | 19 (57.6%) | 14 (42.4%) |  | 33 (100%) |
|  | Missing | 1098 (71.3%) | 442 (28.7%) |  | 1540 (100%) |
| Platelet count (×10^3^/µL) | Thrombocytopenia (<150) | 362 (72.4%) | 138 (27.6%) | 0.384 | 500 (100%) |
|  | Normal (150-450) | 2434 (69.4%) | 1075 (30.6%) |  | 3509 (100%) |
|  | Thrombocytosis (>450) | 50 (69.4%) | 22 (30.6%) |  | 72 (100%) |
|  | Missing | 1075 (71%) | 440 (29%) |  | 1515 (100%) |

Values in cells and parentheses are the number and percentage of patients, respectively.

*Male, 13.8–17.2 g/dL; Female, 12.1–15.1 g/dL

**Male, 41–50%; Female, 36–48%

**Supplementary Table S2.** Characteristics of patients in the external validation cohort

| Variable | Supportive care  (n=324) | Oxygen therapy  (n=82) | Critical care*  (n=39) | *P*-value | Total  (n=445) |
| --- | --- | --- | --- | --- | --- |
| Age (year) | 54 (+/-19) | 73 (+/-14) | 73 (+/-13) | <0.0001 | 59 (+/-20) |
| Male sex | 45.10% | 48.80% | 61.50% | 0.143 | 47.20% |
| Febrile sensation | 33.30% | 34.10% | 52.60% | 0.062 | 35.20% |
| Altered consciousness | 0% | 0% | 7.90% | <0.0001 | 0.70% |
| Shortness of breath | 2.90% | 11% | 42.10% | <0.0001 | 8% |
| Runny nose | 5.60% | 2.40% | 5.30% | 0.509 | 4.90% |
| Hypertension | 32.70% | 68.30% | 63.20% | <0.0001 | 42.30% |
| Diabetes | 14.10% | 35.40% | 39.50% | <0.0001 | 20.40% |
| Kidney disease | 1.60% | 11% | 10.50% | <0.0001 | 4.20% |
| Cancer | 5.60% | 3.70% | 7.90% | 0.617 | 5.40% |
| Dementia | 4.20% | 19.50% | 21.10% | <0.0001 | 8.70% |
| Heart disease | 9.80% | 25.60% | 18.40% | 0.001 | 13.60% |
| Pulse (bpm) | 83 (+/-13) | 84 (+/-17) | 81 (+/-21) | 0.52 | 83 (+/-15) |
| Body temperature (C) | 36.6 (+/-0.5) | 36.7 (+/-0.6) | 37.1 (+/-0.8) | <0.0001 | 36.7 (+/-0.6) |
| White blood cell (n) | 5285 (+/-2139) | 5439 (+/-2724) | 7343 (+/-3521) | <0.0001 | 5497 (+/-2469) |
| Hematocrit (%) | 39.3 (+/-4.6) | 38.2 (+/-5.5) | 36 (+/-5.6) | <0.0001 | 38.8 (+/-5) |
| Platelet (n) | 214053 (+/-69850) | 186561 (+/-82483) | 183308 (+/-86731) | 0.002 | 206187 (+/-74870) |
| Lymphocyte (n) | 1313 (+/-586) | 1009 (+/-528) | 814 (+/-441) | <0.0001 | 1212 (+/-589) |

*the use of a ventilator or extracorporeal membrane oxygenation machine, or died

**Supplementary Table S3.** The full results of predictor selection

|  | **Tier 1** | | | **Tiers 1/2** | | | **Tiers 1/3** | | | **Tiers 1/2/3** | | | **Tiers 1/2/3/4** | | |
| --- | --- | --- | --- | --- | --- | --- | --- | --- | --- | --- | --- | --- | --- | --- | --- |
| **Variable** | OR | Frequency | VI | OR | Frequency | VI | OR | Frequency | VI | OR | Frequency | VI | OR | Frequency | VI |
| Age, 50-59y | 1.062 (0.009) | 100% (500/500) | 24.551 (2.471) | 1.015 (0.009) | 93.6% (468/500) | 13.196 (1.679) | 1.061 (0.009) | 100% (500/500) | 22.811 (2.219) | 1.025 (0.01) | 100% (500/500) | 13.467 (1.476) | 1.002 (0.004) | 32% (160/500) | 13.563 (1.759) |
| Age, 60-69y | 1.19 (0.015) | 100% (500/500) |  | 1.11 (0.015) | 100% (500/500) |  | 1.181 (0.015) | 100% (500/500) |  | 1.113 (0.016) | 100% (500/500) |  | 1.053 (0.016) | 100% (500/500) |  |
| Age, 70-79y | 1.596 (0.037) | 100% (500/500) |  | 1.44 (0.035) | 100% (500/500) |  | 1.599 (0.038) | 100% (500/500) |  | 1.403 (0.033) | 100% (500/500) |  | 1.264 (0.03) | 100% (500/500) |  |
| Age, >=80y | 2.803 (0.122) | 100% (500/500) |  | 2.231 (0.102) | 100% (500/500) |  | 3.034 (0.13) | 100% (500/500) |  | 2.239 (0.111) | 100% (500/500) |  | 1.939 (0.093) | 100% (500/500) |  |
| Male sex | 1.091 (0.01) | 100% (500/500) | 4.405 (1.561) | 1.117 (0.011) | 100% (500/500) | 3.768 (1.611) | 1.103 (0.01) | 100% (500/500) | 5.145 (1.788) | 1.098 (0.011) | 100% (500/500) | 4.507 (1.42) | 1.092 (0.013) | 100% (500/500) | 3.145 (1.308) |
| Pregnancy | 1 (0) | 0% (0/500) | -0.113 (0.431) |  |  |  |  |  |  |  |  |  |  |  |  |
| BMI, underweight | 0.999 (0.003) | 6.8% (34/500) | -0.877 (1.352) |  |  |  |  |  |  |  |  |  |  |  |  |
| BMI, obese | 1.021 (0.011) | 96.4% (482/500) |  |  |  |  |  |  |  |  |  |  |  |  |  |
| Subjective fever | 1.211 (0.014) | 100% (500/500) | 10.094 (2.035) | 1.193 (0.014) | 100% (500/500) | 7.871 (1.785) | 1.096 (0.015) | 100% (500/500) | 7.284 (1.956) | 1.125 (0.017) | 100% (500/500) | 9.073 (1.601) | 1.099 (0.022) | 100% (500/500) | 7.042 (1.681) |
| Cough | 1 (0.001) | 2.2% (11/500) | 0.958 (1.496) |  |  |  |  |  |  |  |  |  |  |  |  |
| Sputum production | 1.004 (0.006) | 54.8% (274/500) | 1.994 (1.445) |  |  |  |  |  |  |  |  |  |  |  |  |
| Sore throat | 0.975 (0.009) | 99.8% (499/500) | 2.311 (1.466) |  |  |  |  |  |  |  |  |  |  |  |  |
| Rhinorrhea | 0.934 (0.01) | 100% (500/500) | 2.808 (1.325) | 0.954 (0.01) | 100% (500/500) | -0.079 (1.367) | 0.932 (0.01) | 100% (500/500) | 1.41 (1.449) |  |  |  |  |  |  |
| Myalgia | 0.999 (0.003) | 16.6% (83/500) | 0.014 (1.439) |  |  |  |  |  |  |  |  |  |  |  |  |
| Fatigue | 1.004 (0.008) | 28% (140/500) | -1.14 (1.311) |  |  |  |  |  |  |  |  |  |  |  |  |
| Dyspnea | 1.665 (0.035) | 100% (500/500) | 19.52 (2.176) | 1.761 (0.04) | 100% (500/500) | 22.778 (2.395) | 1.67 (0.037) | 100% (500/500) | 20.109 (2.22) | 1.678 (0.036) | 100% (500/500) | 14.456 (1.938) | 1.609 (0.036) | 100% (500/500) | 13.531 (1.921) |
| Headache | 0.948 (0.01) | 100% (500/500) | 1.996 (1.48) |  |  |  |  |  |  |  |  |  |  |  |  |
| Altered consciousness | 3.855 (0.462) | 100% (500/500) | 11.742 (1.911) | 2.479 (0.321) | 100% (500/500) | 6.378 (1.722) | 3.375 (0.39) | 100% (500/500) | 10.674 (1.999) | 3.45 (0.474) | 100% (500/500) | 8.738 (1.625) | 3.457 (0.519) | 100% (500/500) | 7.843 (1.371) |
| Nausea or vomiting | 1.005 (0.011) | 26.8% (134/500) | 0.001 (1.41) |  |  |  |  |  |  |  |  |  |  |  |  |
| Diarrhea | 0.999 (0.004) | 16.6% (83/500) | 2.414 (1.514) |  |  |  |  |  |  |  |  |  |  |  |  |
| Diabetes mellitus |  |  |  | 1.148 (0.025) | 100% (500/500) | 1.134 (1.622) |  |  |  | 1.119 (0.023) | 100% (500/500) | 3.741 (1.43) | 1.097 (0.024) | 100% (500/500) | 4.133 (1.426) |
| Hypertension |  |  |  | 1.037 (0.015) | 99.2% (496/500) | 6.05 (1.684) |  |  |  | 1.069 (0.018) | 100% (500/500) | 4.737 (1.408) | 1.055 (0.018) | 99.8% (499/500) | 5.072 (1.569) |
| Heart disease |  |  |  | 1.151 (0.108) | 91.4% (457/500) | 3.581 (1.594) |  |  |  | 1.323 (0.126) | 99.8% (499/500) | 2.809 (1.337) | 1.244 (0.123) | 97.8% (489/500) | 1.319 (1.314) |
| Asthma |  |  |  | 0.992 (0.018) | 31% (155/500) | 1.125 (1.354) |  |  |  |  |  |  |  |  |  |
| COPD |  |  |  | 1.028 (0.05) | 42.6% (213/500) | 1.672 (1.445) |  |  |  |  |  |  |  |  |  |
| Chronic kidney disease |  |  |  | 1.275 (0.11) | 99% (495/500) | 3.068 (1.758) |  |  |  | 1.568 (0.152) | 100% (500/500) | 2.9 (1.524) | 1.666 (0.168) | 100% (500/500) | 2.72 (1.476) |
| Cancer |  |  |  | 1.198 (0.05) | 100% (500/500) | 0.904 (1.419) |  |  |  | 1.166 (0.05) | 100% (500/500) | 0.85 (1.372) | 1.168 (0.046) | 100% (500/500) | 1.756 (1.396) |
| Chronic liver disease |  |  |  | 1.001 (0.009) | 11% (55/500) | 0.589 (1.35) |  |  |  |  |  |  |  |  |  |
| Dementia |  |  |  | 1.464 (0.082) | 100% (500/500) | 6.075 (1.497) |  |  |  | 1.301 (0.077) | 100% (500/500) | 6.261 (1.304) | 1.288 (0.073) | 100% (500/500) | 6.41 (1.472) |
| Autoimmune disease |  |  |  | 1.16 (0.077) | 87.2% (436/500) | 2.153 (1.586) |  |  |  |  |  |  |  |  |  |
| Tachycardia |  |  |  |  |  |  | 1.056 (0.013) | 100% (500/500) | 3.529 (1.663) | 1.052 (0.014) | 100% (500/500) | 0.499 (1.312) | 1.02 (0.014) | 88.4% (442/500) | 1.201 (1.257) |
| Temperature, 37.5-38°C |  |  |  |  |  |  | 1.077 (0.025) | 99.8% (499/500) | 6.974 (1.743) | 1.051 (0.024) | 98.2% (491/500) | 6.489 (1.397) | 1.069 (0.029) | 99.4% (497/500) | 4.267 (1.344) |
| Temperature, 38-38.5°C |  |  |  |  |  |  | 1.384 (0.065) | 100% (500/500) |  | 1.337 (0.064) | 100% (500/500) |  | 1.141 (0.059) | 99.2% (496/500) |  |
| Temperature, >38.5°C |  |  |  |  |  |  | 1.309 (0.075) | 100% (500/500) |  | 1.283 (0.069) | 100% (500/500) |  | 1.134 (0.068) | 97.2% (486/500) |  |
| Systolic BP ≥ 160mmHg |  |  |  |  |  |  | 0.994 (0.01) | 46.6% (233/500) | 1.436 (1.662) | 0.991 (0.013) | 56.6% (283/500) | -0.623 (1.262) |  |  |  |
| Anemia |  |  |  |  |  |  |  |  |  |  |  |  | 1.064 (0.019) | 100% (500/500) | 2.014 (1.38) |
| Thrombocytopenia |  |  |  |  |  |  |  |  |  |  |  |  | 1.133 (0.029) | 100% (500/500) | 2.628 (1.513) |
| Leukocytosis |  |  |  |  |  |  |  |  |  |  |  |  | 1.342 (0.046) | 100% (500/500) | 4.896 (1.504) |
| Lymphocytopenia |  |  |  |  |  |  |  |  |  |  |  |  | 1.452 (0.029) | 100% (500/500) | 13.322 (1.835) |

VI, variable importance; BMI, body mass index; COPD, Chronic obstructive pulmonary disease; BP, blood pressure.

## Supplementary Table S4. Odds ratio of predictors for COVID-19 severity by multivariable ordinal logistic regression in the entire dataset

| **Variable** | | **Univariable** | | **Multivariable with significant**  **variables in univariable analysis** | | **Multivariable with final predictors** | |
| --- | --- | --- | --- | --- | --- | --- | --- |
|  |  | Odds ratio | *p*-value | Odds ratio | *p*-value | Odds ratio | *p*-value |
| Age (years) | <50 | reference |  | reference |  | reference |  |
|  | 50-59 | 4.787 (3.589-6.385) | <0.001 | 2.802 (1.985-3.954) | <0.001 | 2.820 (2.007-3.962) | <0.001 |
|  | 60-69 | 9.264 (7.019-12.227) | <0.001 | 3.954 (2.815-5.555) | <0.001 | 4.057 (2.903-5.67) | <0.001 |
|  | 70-79 | 22.777 (17.15-30.251) | <0.001 | 6.867 (4.775-9.875) | <0.001 | 7.173 (5.024-10.243) | <0.001 |
|  | ≥80 | 60.557 (44.304-82.773) | <0.001 | 15.463 (10.099-23.675) | <0.001 | 16.528 (10.896-25.071) | <0.001 |
| Sex | Female | reference |  |  |  |  |  |
|  | Male | 1.345 (1.159-1.561) | <0.001 | 1.520 (1.234-1.873) | <0.001 | 1.524 (1.244-1.866) | <0.001 |
| BMI (kg/cm^2^) | <23 | reference |  |  |  |  |  |
|  | 23-25 | 1.326 (0.910-1.933) | 0.142 |  |  |  |  |
|  | 25-30 | 1.324 (0.892-1.963) | 0.163 |  |  |  |  |
|  | ≥30 | 1.042 (0.612-1.773) | 0.880 |  |  |  |  |
| Subjective fever | | 2.795 (2.394-3.264) | <0.001 | 1.781 (1.321-2.402) | <0.001 | 1.796 (1.338-2.41) | <0.001 |
| Dyspnea | | 9.017 (7.579-10.727) | <0.001 | 5.241 (4.167-6.591) | <0.001 | 5.712 (4.578-7.127) | <0.001 |
| Altered consciousness | | 51.916 (23.166-116.346) | <0.001 | 11.91 (4.282-33.13) | <0.001 | 13.519 (5.007-36.501) | <0.001 |
| Cough | | 1.281 (1.104-1.486) | 0.001 | 1.062 (0.843-1.338) | 0.611 |  |  |
| Sputum | | 1.394 (1.193-1.630) | <0.001 | 1.229 (0.964-1.567) | 0.096 |  |  |
| Sore throat | | 0.544 (0.428-0.693) | <0.001 | 0.792 (0.577-1.086) | 0.147 |  |  |
| Rhinorrhea | | 0.509 (0.381-0.682) | <0.001 | 0.707 (0.475-1.052) | 0.087 |  |  |
| Fatigue | | 2.042 (1.510-2.762) | <0.001 | 0.985 (0.634-1.53) | 0.945 |  |  |
| Headache | | 0.733 (0.593-0.904) | 0.004 | 0.763 (0.575-1.013) | 0.062 |  |  |
| Nausea or vomiting | | 2.021 (1.505-2.714) | <0.001 | 1.122 (0.761-1.656) | 0.561 |  |  |
| Myalgia | | 1.07 (0.880-1.300) | 0.498 |  |  |  |  |
| Diarrhea | | 1.235 (0.972-1.569) | 0.084 |  |  |  |  |
| Diabetes mellitus | | 3.834 (3.206-4.585) | <0.001 | 1.364 (1.071-1.738) | 0.012 | 1.480 (1.163-1.885) | 0.001 |
| Hypertension | | 4.459 (3.815-5.210) | <0.001 | 1.193 (0.956-1.488) | 0.119 | 1.280 (1.027-1.596) | 0.028 |
| Heart disease | | 9.145 (5.651-14.797) | <0.001 | 1.714 (0.902-3.260) | 0.100 | 1.753 (0.931-3.301) | 0.082 |
| Chronic kidney disease | | 8.653 (5.258-14.239) | <0.001 | 2.351 (1.222-4.523) | 0.010 | 2.443 (1.268-4.706) | 0.008 |
| Cancer | | 2.736 (1.912-3.914) | <0.001 | 1.654 (1.045-2.618) | 0.032 | 1.835 (1.165-2.892) | 0.009 |
| Dementia | | 8.091 (6.198-10.562) | <0.001 | 2.214 (1.541-3.183) | <0.001 | 2.257 (1.577-3.230) | <0.001 |
| Asthma | | 1.844 (1.220-2.787) | 0.004 | 0.911 (0.520-1.596) | 0.744 |  |  |
| COPD | | 5.978 (3.315-10.78) | <0.001 | 1.431 (0.702-2.916) | 0.324 |  |  |
| Chronic liver disease | | 1.989 (1.222-3.237) | 0.006 | 0.835 (0.437-1.594) | 0.584 |  |  |
| Autoimmune disease | | 1.687 (0.802-3.548) | 0.168 |  |  |  |  |
| Body temperature (°C) | <37.5 | reference |  | reference |  | reference |  |
|  | 37.5-38.0 | 2.405 (1.918-3.017) | <0.001 | 1.366 (0.928-2.012) | 0.114 | 1.388 (0.951-2.027) | 0.089 |
|  | 38.0-38.5 | 6.361 (4.544-8.905) | <0.001 | 1.596 (0.937-2.720) | 0.085 | 1.661 (0.989-2.79) | 0.055 |
|  | ≥38.5 | 7.572 (4.974-11.526) | <0.001 | 2.301 (1.281-4.135) | 0.005 | 2.377 (1.329-4.251) | 0.004 |
| Systolic blood pressure (mmHg) | <120 | reference |  | reference |  |  |  |
|  | 120-129 | 0.898 (0.794-1.014) | 0.083 |  |  |  |  |
|  | 130-139 | 0.935 (0.826-1.058) | 0.289 |  |  |  |  |
|  | 140-159 | 1.084 (0.966-1.217) | 0.172 |  |  |  |  |
|  | ≥160 | 1.731 (1.384-2.165) | <0.001 | 0.924 (0.688-1.241) | 0.599 |  |  |
| Diastolic blood pressure (mmHg) | ≥100 | reference |  |  |  |  |  |
|  | 90-99 | 0.945 (0.802-1.113) | 0.496 |  |  |  |  |
|  | 80-89 | 0.934 (0.802-1.087) | 0.377 |  |  |  |  |
|  | <80 | 1.063 (0.915-1.235) | 0.426 |  |  |  |  |
| Heart rate (beat/min) | 60-100 | reference |  | reference |  |  |  |
|  | <60 | 1.288 (0.959-1.730) | 0.093 |  |  |  |  |
|  | ≥100 | 1.406 (1.170-1.689) | <0.001 | 1.163 (0.900-1.504) | 0.248 |  |  |
| Anemia (based on hematocrit) | | 3.178 (2.682-3.766) | <0.001 | 1.379 (1.101-1.727) | 0.005 | 1.424 (1.140-1.780) | 0.002 |
| Thrombocytopenia | | 3.973 (3.266-4.833) | <0.001 | 1.949 (1.516-2.507) | <0.001 | 1.992 (1.554-2.554) | <0.001 |
| Leukocytosis | | 3.800 (2.903-4.973) | <0.001 | 2.924 (2.043-4.184) | <0.001 | 2.905 (2.049-4.119) | <0.001 |
| Lymphocytopenia | | 7.664 (6.437-9.125) | <0.001 | 3.471 (2.806-4.294) | <0.001 | 3.563 (2.887-4.396) | <0.001 |

BMI, body mass index; COPD, chronic obstructive pulmonary disease.

**Supplementary Table S5.** The full results of model performances

| Model | | **No significant treatment Vs. O_2_ therapy or more** | | | | | | | **No critical care required Vs. Critical care* or death** | | | | | | |
| --- | --- | --- | --- | --- | --- | --- | --- | --- | --- | --- | --- | --- | --- | --- | --- |
|  |  | AUC | TP/TN/FP/FN | Sensitivity | Specificity | Accuracy | Precision | NPV | AUC | TP/TN/FP/FN | Sensitivity | Specificity | Accuracy | Precision | NPV |
| OLR | Model1 | 0.880 (0.855-0.904) | 193/1199/236/48 | 80.1% (74.5-84.9) | 83.6% (81.5-85.4) | 83.1% (81.2-84.8) | 45% (40.2-49.8) | 96.2% (94.9-97.1) | 0.903 (0.869-0.937) | 75/1336/251/14 | 84.3% (75-91.1) | 84.2% (82.3-85.9) | 84.2% (82.4-85.9) | 23% (18.5-28) | 99% (98.3-99.4) |
|  | Model2A | 0.889 (0.865-0.912) | 195/1119/209/43 | 81.9% (76.4-86.6) | 84.3% (82.2-86.2) | 83.9% (82-85.7) | 48.3% (43.3-53.3) | 96.3% (95-97.3) | 0.905 (0.869-0.94) | 81/1164/313/8 | 91% (83.1-96) | 78.8% (76.6-80.9) | 79.5% (77.4-81.5) | 20.6% (16.7-24.9) | 99.3% (98.7-99.7) |
|  | Model2B | 0.866 (0.841-0.892) | 181/1147/261/53 | 77.4% (71.4-82.5) | 81.5% (79.3-83.5) | 80.9% (78.9-82.8) | 41% (36.3-45.7) | 95.6% (94.3-96.7) | 0.914 (0.884-0.944) | 72/1312/247/11 | 86.7% (77.5-93.2) | 84.2% (82.2-85.9) | 84.3% (82.4-86) | 22.6% (18.1-27.6) | 99.2% (98.5-99.6) |
|  | Model3 | 0.894 (0.871-0.917) | 192/1082/210/40 | 82.8% (77.3-87.4) | 83.7% (81.6-85.7) | 83.6% (81.6-85.4) | 47.8% (42.8-52.8) | 96.4% (95.2-97.4) | 0.922 (0.892-0.953) | 76/1199/242/7 | 91.6% (83.4-96.5) | 83.2% (81.2-85.1) | 83.7% (81.7-85.5) | 23.9% (19.3-29) | 99.4% (98.8-99.8) |
|  | Model4 | 0.907 (0.884-0.929) | 189/835/172/31 | 85.9% (80.6-90.2) | 82.9% (80.5-85.2) | 83.5% (81.3-85.5) | 52.4% (47.1-57.6) | 96.4% (95-97.6) | 0.927 (0.894-0.96) | 68/1046/100/13 | 84% (74.1-91.2) | 91.3% (89.5-92.8) | 90.8% (89-92.3) | 40.5% (33-48.3) | 98.8% (97.9-99.3) |
| RF | Model1 | 0.793 (0.762-0.825) | 160/1299/136/81 | 66.4% (60-72.3) | 90.5% (88.9-92) | 87.1% (85.4-88.6) | 54.1% (48.2-59.8) | 94.1% (92.8-95.3) | 0.825 (0.776-0.874) | 66/1393/194/23 | 74.2% (63.8-82.9) | 87.8% (86.1-89.3) | 87.1% (85.4-88.6) | 25.4% (20.2-31.1) | 98.4% (97.6-99) |
|  | Model2A | 0.843 (0.813-0.872) | 187/1130/198/51 | 78.6% (72.8-83.6) | 85.1% (83.1-87) | 84.1% (82.2-85.9) | 48.6% (43.5-53.7) | 95.7% (94.4-96.8) | 0.858 (0.812-0.904) | 68/1321/156/21 | 76.4% (66.2-84.8) | 89.4% (87.8-91) | 88.7% (87-90.2) | 30.4% (24.4-36.8) | 98.4% (97.6-99) |
|  | Model2B | 0.806 (0.774-0.838) | 167/1210/198/67 | 71.4% (65.1-77.1) | 85.9% (84-87.7) | 83.9% (82-85.6) | 45.8% (40.6-51) | 94.8% (93.4-95.9) | 0.863 (0.819-0.908) | 69/1348/211/14 | 83.1% (73.3-90.5) | 86.5% (84.7-88.1) | 86.3% (84.5-87.9) | 24.6% (19.7-30.1) | 99% (98.3-99.4) |
|  | Model3 | 0.86 (0.833-0.888) | 194/1051/241/38 | 83.6% (78.2-88.1) | 81.3% (79.1-83.4) | 81.7% (79.7-83.6) | 44.6% (39.9-49.4) | 96.5% (95.2-97.5) | 0.91 (0.876-0.944) | 74/1206/235/9 | 89.2% (80.4-94.9) | 83.7% (81.7-85.6) | 84% (82.1-85.8) | 23.9% (19.3-29.1) | 99.3% (98.6-99.7) |
|  | Model4 | 0.884 (0.857-0.911) | 190/799/208/30 | 86.4% (81.1-90.6) | 79.3% (76.7-81.8) | 80.6% (78.3-82.8) | 47.7% (42.7-52.8) | 96.4% (94.9-97.5) | 0.925 (0.892-0.958) | 69/1017/129/12 | 85.2% (75.6-92.1) | 88.7% (86.8-90.5) | 88.5% (86.6-90.2) | 34.8% (28.2-41.9) | 98.8% (98-99.4) |
| Linear SVM | Model1 | 0.851 (0.825-0.877) | 179/1142/292/62 | 74.3% (68.3-79.7) | 79.6% (77.5-81.7) | 78.9% (76.8-80.8) | 38% (33.6-42.6) | 94.9% (93.4-96) | 0.898 (0.863-0.932) | 80/1210/376/9 | 89.9% (81.7-95.3) | 76.3% (74.1-78.4) | 77% (74.9-79) | 17.5% (14.2-21.4) | 99.3% (98.6-99.7) |
|  | Model2A | 0.857 (0.832-0.883) | 179/1086/252/58 | 75.5% (69.5-80.9) | 81.2% (79-83.2) | 80.3% (78.3-82.3) | 41.5% (36.8-46.3) | 94.9% (93.5-96.1) | 0.896 (0.86-0.931) | 78/1191/295/11 | 87.6% (79-93.7) | 80.1% (78-82.1) | 80.6% (78.5-82.5) | 20.9% (16.9-25.4) | 99.1% (98.4-99.5) |
|  | Model2B | 0.859 (0.832-0.885) | 179/1151/257/55 | 76.5% (70.5-81.8) | 81.7% (79.6-83.7) | 81% (79-82.9) | 41.1% (36.4-45.8) | 95.4% (94.1-96.5) | 0.91 (0.879-0.941) | 73/1306/253/10 | 88% (79-94.1) | 83.8% (81.8-85.6) | 84% (82.1-85.7) | 22.4% (18-27.3) | 99.2% (98.6-99.6) |
|  | Model3 | 0.857 (0.83-0.883) | 163/1149/163/67 | 70.9% (64.5-76.7) | 87.6% (85.7-89.3) | 85.1% (83.2-86.8) | 50% (44.4-55.6) | 94.5% (93.1-95.7) | 0.913 (0.882-0.944) | 76/1180/279/7 | 91.6% (83.4-96.5) | 80.9% (78.8-82.9) | 81.5% (79.4-83.4) | 21.4% (17.3-26) | 99.4% (98.8-99.8) |
|  | Model4 | 0.87 (0.842-0.897) | 169/821/191/46 | 78.6% (72.5-83.9) | 81.1% (78.6-83.5) | 80.7% (78.4-82.9) | 46.9% (41.7-52.2) | 94.7% (93-96.1) | 0.921 (0.888-0.955) | 68/1011/135/13 | 84% (74.1-91.2) | 88.2% (86.2-90) | 87.9% (86-89.7) | 33.5% (27-40.4) | 98.7% (97.8-99.3) |
| RBF SVM | Model1 | 0.625 (0.577-0.673) | 105/1363/71/136 | 43.6% (37.2-50.1) | 95% (93.8-96.1) | 87.6% (86-89.2) | 59.7% (52-67) | 90.9% (89.4-92.3) | 0.863 (0.819-0.907) | 75/1260/326/14 | 84.3% (75-91.1) | 79.4% (77.4-81.4) | 79.7% (77.7-81.6) | 18.7% (15-22.9) | 98.9% (98.2-99.4) |
|  | Model2A | 0.727 (0.684-0.771) | 142/1189/149/95 | 59.9% (53.4-66.2) | 88.9% (87.1-90.5) | 84.5% (82.6-86.3) | 48.8% (42.9-54.7) | 92.6% (91-94) | 0.877 (0.836-0.919) | 76/1228/258/13 | 85.4% (76.3-92) | 82.6% (80.6-84.5) | 82.8% (80.8-84.6) | 22.8% (18.4-27.6) | 99% (98.2-99.4) |
|  | Model2B | 0.759 (0.72-0.799) | 134/1248/160/100 | 57.3% (50.7-63.7) | 88.6% (86.9-90.2) | 84.2% (82.3-85.9) | 45.6% (39.8-51.5) | 92.6% (91.1-93.9) | 0.879 (0.837-0.922) | 71/1329/230/12 | 85.5% (76.1-92.3) | 85.2% (83.4-87) | 85.3% (83.5-86.9) | 23.6% (18.9-28.8) | 99.1% (98.4-99.5) |
|  | Model3 | 0.796 (0.761-0.831) | 158/1140/172/72 | 68.7% (62.3-74.6) | 86.9% (84.9-88.7) | 84.2% (82.3-86) | 47.9% (42.4-53.4) | 94.1% (92.6-95.3) | 0.906 (0.873-0.94) | 77/1201/258/6 | 92.8% (84.9-97.3) | 82.3% (80.3-84.2) | 82.9% (80.9-84.7) | 23% (18.6-27.9) | 99.5% (98.9-99.8) |
|  | Model4 | 0.813 (0.773-0.853) | 162/830/182/53 | 75.3% (69-81) | 82% (79.5-84.3) | 80.8% (78.5-83) | 47.1% (41.7-52.5) | 94% (92.2-95.5) | 0.903 (0.86-0.946) | 70/986/160/11 | 86.4% (77-93) | 86% (83.9-88) | 86.1% (84-88) | 30.4% (24.6-36.8) | 98.9% (98-99.4) |
| KMA model | | 0.723 (0.693-0.753) | 129/108/1308/106 | 54.9% (61.4) | 7.6% (6.3-9.148.3-) | 14.4% (12.7-16.1) | 9% (7.5-10.6) | 50.5% (43.6-57.4) | 0.728 (0.678-0.778) | 43/1395/171/42 | 50.6% (39.5-61.6) | 89.1% (87.4-90.6) | 87.1% (85.4-88.7) | 20.1% (14.9-26.1) | 97.1% (96.1-97.9) |
| MEWS | | 0.598 (0.563-0.633) | 129/314/1023/98 | 56.8% (50.1-63.4) | 23.5% (21.2-25.9) | 28.3% (26.1-30.6) | 11.2% (9.4-13.2) | 76.2% (71.8-80.2) | 0.631 (0.574-0.689) | 41/1112/371/40 | 50.6% (39.3-61.9) | 75% (72.7-77.2) | 73.7% (71.5-75.9) | 10% (7.2-13.3) | 96.5% (95.3-97.5) |

Values in parentheses are 95% confidence intervals. OLR, ordinal logistic regression; RF, random forest; SVM, support vector machine; RBF, radial basis function; KMA, Korean Medical Association; MEWS, Modified Early Warning Score; AUC, area under the receiver operator characteristics curve; TP, true positive; TN, true negative, FP, false positive, FN, false negative; NPV, negative predictive value.

*multi-organ failure, the use of a ventilator or extracorporeal membrane oxygenation machine.

## Supplementary Table S6. Model performances by cutoff probabilities in the internal validation cohort

| **Model** | **Cutoff** | **No significant treatment Vs. O_2_ therapy or more** | | | | | | **No critical care required Vs. Critical care* or death** | | | | | |
| --- | --- | --- | --- | --- | --- | --- | --- | --- | --- | --- | --- | --- | --- |
|  |  | TP/TN/FP/FN | Sensitivity | Specificity | Accuracy | Precision | NPV | TP/TN/FP/FN | Sensitivity | Specificity | Accuracy | Precision | NPV |
| **Model 1** | 5% | 227/714/721/14 | 94.2% (90.4-96.8) | 49.8% (47.1-52.4) | 56.1% (53.7-58.5) | 23.9% (21.3-26.8) | 98.1% (96.8-98.9) | 76/1295/292/13 | 85.4% (76.3-92) | 81.6% (79.6-83.5) | 81.8% (79.9-83.6) | 20.7% (16.6-25.2) | 99% (98.3-99.5) |
|  | 10% | 208/1090/345/33 | 86.3% (81.3-90.4) | 76% (73.7-78.1) | 77.4% (75.4-79.4) | 37.6% (33.6-41.8) | 97.1% (95.9-98) | 69/1408/179/20 | 77.5% (67.4-85.7) | 88.7% (87.1-90.2) | 88.1% (86.5-89.6) | 27.8% (22.3-33.8) | 98.6% (97.8-99.1) |
|  | 15% | 207/1107/328/34 | 85.9% (80.8-90) | 77.1% (74.9-79.3) | 78.4% (76.4-80.3) | 38.7% (34.5-43) | 97% (95.9-97.9) | 51/1492/95/38 | 57.3% (46.4-67.7) | 94% (92.7-95.1) | 92.1% (90.7-93.3) | 34.9% (27.2-43.3) | 97.5% (96.6-98.2) |
|  | 20% | 178/1237/198/63 | 73.9% (67.8-79.3) | 86.2% (84.3-87.9) | 84.4% (82.6-86.1) | 47.3% (42.2-52.5) | 95.2% (93.8-96.3) | 44/1511/76/45 | 49.4% (38.7-60.2) | 95.2% (94-96.2) | 92.8% (91.4-94) | 36.7% (28.1-45.9) | 97.1% (96.1-97.9) |
|  | 25% | 168/1277/158/73 | 69.7% (63.5-75.4) | 89% (87.3-90.6) | 86.2% (84.5-87.8) | 51.5% (46-57.1) | 94.6% (93.2-95.7) | 32/1544/43/57 | 36% (26.1-46.8) | 97.3% (96.4-98) | 94% (92.8-95.1) | 42.7% (31.3-54.6) | 96.4% (95.4-97.3) |
| **Model 2A** | 5% | 224/711/618/14 | 94.1% (90.3-96.7) | 53.5% (50.8-56.2) | 59.7% (57.2-62.1) | 26.6% (23.6-29.7) | 98.1% (96.8-98.9) | 79/1184/294/10 | 88.8% (80.3-94.5) | 80.1% (78-82.1) | 80.6% (78.6-82.5) | 21.2% (17.1-25.7) | 99.2% (98.5-99.6) |
|  | 10% | 212/944/385/26 | 89.1% (84.4-92.7) | 71% (68.5-73.5) | 73.8% (71.5-75.9) | 35.5% (31.7-39.5) | 97.3% (96.1-98.2) | 69/1328/150/20 | 77.5% (67.4-85.7) | 89.9% (88.2-91.3) | 89.2% (87.5-90.6) | 31.5% (25.4-38.1) | 98.5% (97.7-99.1) |
|  | 15% | 202/1067/262/36 | 84.9% (79.7-89.2) | 80.3% (78-82.4) | 81% (79-82.9) | 43.5% (39-48.2) | 96.7% (95.5-97.7) | 58/1379/99/31 | 65.2% (54.3-75) | 93.3% (91.9-94.5) | 91.7% (90.2-93) | 36.9% (29.4-45) | 97.8% (96.9-98.5) |
|  | 20% | 192/1130/199/46 | 80.7% (75.1-85.5) | 85% (83-86.9) | 84.4% (82.5-86.1) | 49.1% (44-54.2) | 96.1% (94.8-97.1) | 45/1416/62/44 | 50.6% (39.8-61.3) | 95.8% (94.7-96.8) | 93.2% (91.9-94.4) | 42.1% (32.6-52) | 97% (96-97.8) |
|  | 25% | 168/1198/131/70 | 70.6% (64.4-76.3) | 90.1% (88.4-91.7) | 87.2% (85.4-88.8) | 56.2% (50.4-61.9) | 94.5% (93.1-95.7) | 40/1431/47/49 | 44.9% (34.4-55.9) | 96.8% (95.8-97.7) | 93.9% (92.6-95) | 46% (35.2-57) | 96.7% (95.6-97.5) |
| **Model 2B** | 5% | 254/4317/858/33 | 88.5% (84.2-92) | 83.4% (82.4-84.4) | 83.7% (82.7-84.7) | 22.8% (20.4-25.4) | 99.2% (98.9-99.5) | 736/2680/1986/60 | 92.5% (90.4-94.2) | 57.4% (56-58.9) | 62.5% (61.2-63.8) | 27% (25.4-28.7) | 97.8% (97.2-98.3) |
|  | 10% | 225/4676/499/62 | 78.4% (73.2-83) | 90.4% (89.5-91.1) | 89.7% (88.9-90.5) | 31.1% (27.7-34.6) | 98.7% (98.3-99) | 670/3489/1177/126 | 84.2% (81.4-86.6) | 74.8% (73.5-76) | 76.1% (75-77.3) | 36.3% (34.1-38.5) | 96.5% (95.9-97.1) |
|  | 15% | 181/4861/314/106 | 63.1% (57.2-68.7) | 93.9% (93.2-94.6) | 92.3% (91.6-93) | 36.6% (32.3-41) | 97.9% (97.4-98.2) | 620/3817/849/176 | 77.9% (74.8-80.7) | 81.8% (80.7-82.9) | 81.2% (80.2-82.3) | 42.2% (39.7-44.8) | 95.6% (94.9-96.2) |
|  | 20% | 159/4940/235/128 | 55.4% (49.4-61.2) | 95.5% (94.9-96) | 93.4% (92.7-94) | 40.4% (35.5-45.4) | 97.5% (97-97.9) | 569/4059/607/227 | 71.5% (68.2-74.6) | 87% (86-87.9) | 84.7% (83.7-85.7) | 48.4% (45.5-51.3) | 94.7% (94-95.4) |
|  | 25% | 126/5029/146/161 | 43.9% (38.1-49.9) | 97.2% (96.7-97.6) | 94.4% (93.7-95) | 46.3% (40.3-52.4) | 96.9% (96.4-97.4) | 531/4192/474/265 | 66.7% (63.3-70) | 89.8% (88.9-90.7) | 86.5% (85.5-87.4) | 52.8% (49.7-56) | 94.1% (93.3-94.7) |
| **Model 3** | 5% | 219/694/598/13 | 94.4% (90.6-97) | 53.7% (51-56.5) | 59.9% (57.4-62.4) | 26.8% (23.8-30) | 98.2% (96.9-99) | 76/1182/259/7 | 91.6% (83.4-96.5) | 82% (79.9-84) | 82.5% (80.5-84.4) | 22.7% (18.3-27.6) | 99.4% (98.8-99.8) |
|  | 10% | 209/912/380/23 | 90.1% (85.5-93.6) | 70.6% (68-73.1) | 73.6% (71.3-75.8) | 35.5% (31.6-39.5) | 97.5% (96.3-98.4) | 63/1296/145/20 | 75.9% (65.3-84.6) | 89.9% (88.3-91.4) | 89.2% (87.5-90.7) | 30.3% (24.1-37) | 98.5% (97.7-99.1) |
|  | 15% | 196/1045/247/36 | 84.5% (79.2-88.9) | 80.9% (78.6-83) | 81.4% (79.4-83.4) | 44.2% (39.6-49) | 96.7% (95.4-97.7) | 54/1349/92/29 | 65.1% (53.8-75.2) | 93.6% (92.2-94.8) | 92.1% (90.6-93.4) | 37% (29.2-45.4) | 97.9% (97-98.6) |
|  | 20% | 184/1114/178/48 | 79.3% (73.5-84.3) | 86.2% (84.2-88.1) | 85.2% (83.3-86.9) | 50.8% (45.6-56.1) | 95.9% (94.6-96.9) | 44/1385/56/39 | 53% (41.7-64.1) | 96.1% (95-97.1) | 93.8% (92.4-94.9) | 44% (34.1-54.3) | 97.3% (96.3-98) |
|  | 25% | 168/1156/136/64 | 72.4% (66.2-78.1) | 89.5% (87.7-91.1) | 86.9% (85.1-88.5) | 55.3% (49.5-60.9) | 94.8% (93.4-95.9) | 38/1398/43/45 | 45.8% (34.8-57.1) | 97% (96-97.8) | 94.2% (92.9-95.3) | 46.9% (35.7-58.3) | 96.9% (95.8-97.7) |
| **Model 4** | 5% | 212/468/539/8 | 96.4% (93-98.4) | 46.5% (43.4-49.6) | 55.4% (52.6-58.2) | 28.2% (25-31.6) | 98.3% (96.7-99.3) | 71/936/210/10 | 87.7% (78.5-93.9) | 81.7% (79.3-83.9) | 82.1% (79.8-84.2) | 25.3% (20.3-30.8) | 98.9% (98.1-99.5) |
|  | 10% | 202/693/314/18 | 91.8% (87.4-95.1) | 68.8% (65.9-71.7) | 72.9% (70.4-75.4) | 39.1% (34.9-43.5) | 97.5% (96-98.5) | 68/1039/107/13 | 84% (74.1-91.2) | 90.7% (88.8-92.3) | 90.2% (88.4-91.8) | 38.9% (31.6-46.5) | 98.8% (97.9-99.3) |
|  | 15% | 190/807/200/30 | 86.4% (81.1-90.6) | 80.1% (77.5-82.6) | 81.3% (79-83.4) | 48.7% (43.7-53.8) | 96.4% (94.9-97.6) | 62/1068/78/19 | 76.5% (65.8-85.2) | 93.2% (91.6-94.6) | 92.1% (90.4-93.5) | 44.3% (35.9-52.9) | 98.3% (97.3-98.9) |
|  | 20% | 178/862/145/42 | 80.9% (75.1-85.9) | 85.6% (83.3-87.7) | 84.8% (82.6-86.7) | 55.1% (49.5-60.6) | 95.4% (93.8-96.6) | 55/1094/52/26 | 67.9% (56.6-77.8) | 95.5% (94.1-96.6) | 93.6% (92.1-94.9) | 51.4% (41.5-61.2) | 97.7% (96.6-98.5) |
|  | 25% | 162/896/111/58 | 73.6% (67.3-79.3) | 89% (86.9-90.8) | 86.2% (84.2-88.1) | 59.3% (53.3-65.2) | 93.9% (92.2-95.4) | 48/1110/36/33 | 59.3% (47.8-70.1) | 96.9% (95.7-97.8) | 94.4% (92.9-95.6) | 57.1% (45.9-67.9) | 97.1% (96-98) |

*multi-organ failure, the use of a ventilator or extracorporeal membrane oxygenation machine

TP, true positive; TN, true negative, FP, false positive, FN, false negative; NPV, negative predictive value

**Supplementary Table S7.** Criteria for COVID-19 severity proposed by the Korea Medical Association

| **Severity** | **Criteria** |
| --- | --- |
| **Asymptomatic** | All of the following conditions must be met:  1: No altered consciousness  2: Younger than 50 years  3: No underlying disease  4: Temperature <37.5°C without taking antipyretics  5: No smoking |
| **Mild** | No altered consciousness AND at least one of the following conditions:  1: Younger than 50 years  2: No underlying disease  3: Temperature ≤38°C while taking antipyretics  4: More than one relevant symptom* except for altered consciousness  5: Smoking |
| **Moderate** | No altered consciousness AND at least one of the following conditions:  1: Temperature >38°C while taking antipyretics  2: Shortness of breath (dyspnea)  3: Radiologic evidence of pneumonia |
| **Severe** | At least one of the following conditions:  1: Altered consciousness  2: Moderate or severe shortness of breath (dyspnea)  3: Oxygen saturation ≤ 90%  4: Radiologic evidence of moderate pneumonia involving lungs bilaterally or >50% |

If a patient is from a long-term care facility or facility for the handicapped, disease severity should be upgraded by one level. Forty-eight hours after the relevant symptoms are resolved, disease severity can be downgraded by one level.

*headache, cough, sore throat, sputum production, fatigue, myalgia, shortness of breath (dyspnea)

**Table S8.** Modified Early Warning Score (MEWS)

| **Score** | **3** | **2** | **1** | **0** | **1** | **2** | **3** |
| --- | --- | --- | --- | --- | --- | --- | --- |
| Respiratory rate (per min) |  | ≤ 8 |  | 9–14 | 15–20 | 21–29 | > 29 |
| Heart rate (per min) |  | ≤ 40 | 41–50 | 51–100 | 101–110 | 111–129 | > 129 |
| Systolic BP (mmHg) | ≤ 70 | 71–80 | 81–100 | 101–199 |  | ≥ 200 |  |
| Temperature (°C) |  | ≤ 35 | 35.1–36 | 36.1–38 | 38.1–38.5 | ≥ 38.6 |  |
| Neurological |  |  |  | Alert | Reacting to voice | Reacting to pain | Unresponsive |

According to the sum of the scores, disease severity can be determined as follows: 0-4, mild (low risk); 5-6, moderate (intermediate risk); 7 or higher, severe (high risk). If the total score is 7 or higher (high risk), consider applying critical care.
